# Correlation Between Proprioceptive Impairment and Motor Deficits After Stroke: A Meta-Analysis Review

**DOI:** 10.3389/fneur.2021.688616

**Published:** 2022-01-13

**Authors:** Yifan Yu, Yufang Chen, Teng Lou, Xia Shen

**Affiliations:** ^1^Department of Physical Therapy, Shanghai YangZhi Rehabilitation Hospital (Shanghai Sunshine Rehabilitation Center), School of Medicine, Tongji University, Shanghai, China; ^2^Laboratory of Biomechanics and Rehabilitation Engineering, School of Medicine, Tongji University, Shanghai, China; ^3^Rehabilitation center, Shanghai First Rehabilitation Hospital, Shanghai, China; ^4^Rehabilitation Medicine Research Center, Shanghai YangZhi Rehabilitation Hospital (Shanghai Sunshine Rehabilitation Center), School of Medicine, Tongji University, Shanghai, China; ^5^Department of Rehabilitation Sciences, School of Medicine, Tongji University, Shanghai, China

**Keywords:** stroke, proprioception, motor function, rehabilitation, ICF

## Abstract

**Introduction:** Proprioceptive impairment is a common symptom after stroke. Clarifying how proprioception correlates with motor function after stroke may be helpful in optimizing proprioception-augmented movement training. Previous studies have shown inconsistent findings. A meta-analysis is an optimal method to explore the correlation and identify the factors contributing to these inconsistencies.

**Objective:** To explore the correlation between proprioception and motor function after stroke through a meta-analysis, taking into account characteristics of the measurements used in these studies.

**Methods:** We searched multiple databases until November 2021 for eligible studies that measured both proprioception and motor functions in persons with stroke and reported their correlation or data for correlation analysis. A meta-analysis of the correlations was performed. The subgroup analysis and meta-regression were further conducted to investigate potential factors contributing to the heterogeneity of correlation strength, based on the participants' characteristics, proprioception, and motor function measures.

**Results:** In total, 28 studies comprising of 1,829 participants with stroke were included in the meta-analysis. The overall correlation between proprioception and motor function was significant (*r* = 0.267, *p* < 0.05), but there was heterogeneity across studies (*I*^2^ = 45%, *p* < 0.05). The results of the subgroup analysis showed proprioception of the axial segment in weight-bearing conditions (*r* = 0.443, *p* < 0.05) and upper limb without weight-bearing (*r* = 0.292, *p* < 0.05) had a stronger correlation with motor function than proprioception of the lower limb without weight-bearing. The proprioception measured through ipsilateral matching (*r* = 0.412, *p* < 0.05) showed a stronger correlation with motor function than through contralateral matching. The International Classification of Functioning, Disability, and Health (ICF) domains of motor function, movement function (*r* = 0.338, *p* < 0.05), activity performance (*r* = 0.239, *p* < 0.05), and independence (*r* = 0.319, *p* < 0.05) showed a stronger correlation with proprioception than with other domains.

**Conclusion:** There is a significant correlation between proprioception and motor dysfunction after stroke. The proprioception measured in the axial segment under weight-bearing conditions or measured with ipsilateral matching, and motor function, specifically in the ICF domains of movement function, activity performance, and independence showed a positive contribution to the association between proprioception and motor function. The correlation does not imply causation and might be underestimated by attributes of current tests for proprioception and motor function. Further studies are needed to clarify the cause-effect relationship.

## Introduction

Proprioception is the sense of position and motion of one's own body parts and the force generated during movement (1, 2). It is essential for motor control during movement, balance, and locomotive tasks, which provides feedforward information for motor planning and delivers rapid feedback information upward for motor response and/or adaptation. With its important role in upward feedback mechanisms, proprioception is crucial for motor learning, especially in skill refinement (2, 3).

Proprioceptive deficits may present when damage occurs in the proprioceptive receptors (4), any part of the afferent pathways (5), or the cortex area responsible for the sensory integration and perception (2, 6). These deficits are common after stroke, resulting from cerebral injury, as well as secondary dysfunction of afferent pathways, and proprioceptive receptors due to motor paralysis (7, 8). The prevalence rate of proprioceptive deficits is as high as 54–64% in persons with stroke (7, 8). The condition of proprioception has become an important evaluation item in the rehabilitation management of persons with stroke (1, 9). Some clinicians and researchers have further explored the correlation between proprioception and motor function after stroke, aiming to clarify the influence of proprioceptive deficits on motor function (9–14). However, the results were inconsistent across studies, with only some of them finding significant correlation (9–14).

The diverse settings of proprioception or motor function measurements could be the main cause of inconsistent findings on the correlation between proprioception and motor function after the stroke across studies. Owing to the complexity of the pathological processes of proprioceptive deficits, various methods have been developed to measure proprioceptive acuity (1), such as the thumb localizing test (TLT) (15), position or movement matching test at different joints (11–13), and the robotic exoskeleton-assisted arm position test (14). Any divergence in testing characteristics, such as subtypes of proprioception targeted, body part targeted, testing task, or accuracy of results could be a potential reason for the different results in the correlation between proprioception and motor function (12). For motor function, various domains measured in previous studies, such as muscle tension (12), movement function of the upper limb (14) or lower limbs (13), activity performance (10), or independence (9, 11) could be other key factors contributing to the incoherent findings on their correlation with proprioception after stroke. Considering these characteristics of proprioception and motor function, it is essential to explore the correlation between proprioception and motor function after stroke. Clarification of their correlation in this specific manner should be useful in providing explicit evidence for designing proprioception-augmented rehabilitation training. However, it is difficult to design an original correlation study to include all the aforementioned characteristics of proprioception and motor function measures. Meta-analysis is the optimal method, but previous studies using this method have been lacking.

This study aimed to investigate the correlation between proprioceptive impairment and motor deficits after stroke through a meta-analysis, taking the characteristics of measures into account. We hypothesized that proprioception and motor function are correlated in persons with stroke, and characteristics of proprioception or motor function might have different influences on the correlation.

## Methods

### Identification and Selection of Studies

The review was registered on the PROSPERO International System Evaluation Prospective Registration website (registration number: CRD42020184181) in May 2020 and was conducted according to the “Preferred Reporting Project for Systematic Evaluation and Meta-Analysis” (PRISMA). Databases, including Web of Science, CINAHL complete, SportDiscus, MEDLINE, and Academic Search Premier through EbscoHost, were searched until November 15, 2021. There was no limit regarding publication dates but restricted to English language articles. The search strategies related to stroke, proprioception, and motor function were used (propriocep^*^ OR position sense OR movement sense OR velocity sense OR force sense) AND (stroke OR hemiplegia OR cerebrovascular accident) AND (Motor function OR balance function OR mobility) AND (Relation^*^ OR correlation OR effect OR difference).

All potential articles were imported into Endnote X9 (Clarivate, Philadelphia, PA, USA). The duplicates were then removed. Titles and abstracts were screened by three reviewers (YFY, YFC, and TL). When the abstract of an article suggested that it might meet the inclusion criteria, the full text was read to check its eligibility, and it was included if it fulfilled the selection criteria. The reference lists of the relevant articles were searched as additional sources. The corresponding author (XS) was consulted when there was a disagreement.

### Inclusion and Exclusion Criteria

Studies identified through the database search were evaluated by reviewers to ensure that the study met all of the following inclusion criteria:

1. targeted at adults with stroke.
2. included proprioception measures with a clear description and interpretation of the measurements and results.
3. included motor function measures with a clear description and interpretation of the measurements and results.
4. presented the results of the correlation between proprioception and motor function; the presented data of each function were sufficient to analyze their correlation.

Studies were excluded when the following criteria were not met:

1. The correlation was explored only after interventions.
2. The description of proprioception or motor function measures was not sufficiently clear to interpret their correlation (16).

### Assessment of Methodological Quality

Quality assessment of diagnostic accuracy studies scale-second version (QUADAS-2) was adopted to evaluate the methodological quality of the included studies. QUADAS-2 is the most recommended tool to date to evaluate the risk of bias in diagnostic accuracy studies (17) and association studies (18). QUADAS-2 consists of four key domains: person selection, index test, reference standard, and flow and timing. Each key domain has a set of signaling questions to help reach judgments regarding the risk of bias.

To ensure consistent assessments, a rating guideline of QUADAS-2 with detailed criteria was developed by reviewers with tailored signaling questions according to the research question of this study (Table 1). Each signaling question was answered as “yes,” “no,” or “unclear.” Three reviewers (YFY, YFC, and TL) independently assessed the methodological quality of the included studies. Disagreement on the scoring was discussed with the corresponding author and resolved.

**Table 1:** Tailed scoring guideline of quality assessment of diagnostic accuracy studies scale (QUADAS).

**Risk of bias**	
**Signaling questions**	**Tailed signaling questions**
***Patient selection***	
1) Was a consecutive or random sample of patients enrolled?	1) If patients with stroke were recruited based on a consecutive series or a random sample, score this item as “Yes.” When patients were enrolled with convenience sampling or other non-probability sampling method, then score as “No.” If no sampling information was given by authors, score as “Unclear”
2) Was a case-control design avoided?	2) This research study aimed to clarify the correlation between proprioceptive impairment and motor dysfunction in stroke with patients. If there was no selection criteria of known condition of proprioceptive impairment, score this item as “Yes,” otherwise, scored as “No.” If no information was given by authors, score as “Unclear.”
3) Did the study avoid inappropriate exclusions?	3) If the study has no criteria to exclude some subjects who had some proprioception conditions or some motor impairment, such as the best level or the poorest level, etc., score this item as “Yes,” otherwise score as “No.” If no information was given by authors, score as “Unclear.”
**Index test**	
1) Were the index test results interpreted without knowledge of the results of the reference standard?	2) The targets of index test and reference test in our study were proprioception and motor function, respectively. If the proprioception test was conducted and interpreted without knowing the results of the motor function test, or if the proprioception and motor function were tested at the same time node, score this item as “Yes,” otherwise scored as “No.” If no information was given by authors, score as “Unclear.”
2) If a threshold was used, was it pre-specified?	3) If no threshold of proprioception test was used to scale the level of impairment, or if a threshold of index test was used and pre-specified, score this item as “Yes,” otherwise score as “No.” If no information was given by authors, score as “Unclear.”
**Reference standard**	
1) Is the reference standard likely to correctly classify the target condition?	2) The reference test in our study targeted the motor function. If the method can assess the motor function correctly, score this item as “Yes.” If the assessment were conducted incorrectly, score as “No.” If no detailed information of reference test was given by authors, score this item as “Unclear.”
2) Were the reference standard results interpreted without knowledge of the results of the index test?	3) If the proprioception test was conducted and interpreted without knowing the results of motor function test, or if the proprioceptive and motor functions were tested at the same time node, score this item as “Yes.” Otherwise, score as “No.” If no information was given by authors, score as “Unclear.”
**Flow and timing**	
1) Was there an appropriate interval between index test(s) and reference standard?	1) If the proprioceptive and motor functions were tested at the same time node, score this item as “Yes,” otherwise, score as “No.” If no information was given by authors, score as “Unclear.”
2) Did all patients receive a reference standard?	2) If all patients received motor function tests, score this item as “Yes,” If not all patients received motor function tests, score this item as “No,” If no relevant information was presented, score as “Unclear.”
3) Did patients receive the same reference standard?	3) If motor function tests were the same for all patients, scored this item as “Yes,” otherwise score as “No.” If no information was given by authors, score as “Unclear.”
4) Were all patients included in the analysis?	4) If all patients were included in the analysis, or if either proprioception score or motor function assessment of any patients in the study was not reported but authors provided a valid explanation, score this as “Yes,” otherwise, score as “No.” If no information was given by authors, score as “Unclear.”

### Data Extraction

The extracted data consisted of the author (year), sample size, characteristics of participants, proprioception, and motor function measures, and correlation between proprioception and motor function in the form of correlation coefficients or *p*. The correlation coefficients were extracted from the published data of most of the included studies or by analyzing the original published data of several studies using the Pearson correlation test (19–22). The characteristics of participants extracted included age, sex, time after stroke onset, affected side of hemisphere region, stroke type, and spatial neglect.

For proprioception measures, the following characteristics were extracted from the studies, including subtypes of proprioception tested, test region and position, matching side, matching movement, number of joint planes of task movements, and result data type.

Proprioception generally has three subtypes which are position, motion, and force senses. Position sense refers to the ability to perceive the position of a joint or body part. Motion sense is the ability to identify the movement speed or direction of a joint or body part. Force sense is the ability to recognize the force of the muscles or joints (1, 2). The force sense had not been tested in the included studies, and, thus, was not further described in the rest of the “Method” and “Result” sections.

Proprioception is usually measured at limbs and trunks in weight-bearing or non-weight-bearing positions. For weight-bearing conditions, standing and sitting are common positions during which the trunk and lower limb are in the midline region of the body, thereby named as axial body segment. So, test regions and positions were categorized as “the upper limb or lower limb without weight bearing,” and “the axial segment in weight-bearing position.”

Matching is the commonly-used task for measuring proprioception. It usually consists of two main steps, first generating a reference position or movement, then matching the reference position or movement. Based on the body part involved in the two steps of movement, the task has two kinds, “ipsilateral” and “contralateral.” Ipsilateral matching indicates that the body part (limb or axial segment) involved in reference-matching movement is the same as that in the reference-generating movement, while contralateral matching implies the limbs for reference generation and replication are on the opposite side. The movement of the matching task has two modes, “active” or “passive.” We described the mode of movement at each step of the matching task. For some tests, reference matching is just performed perceptually without movement, such as by participants reporting the position perceived during the first reference movement. The matching of these tests was described as “ipsilateral” type and with “perceptual” mode.

The joint plane includes frontal, sagittal, and transverse types. The number of joint planes was recorded as “single” when one plane at one joint was involved, such as knee flexion-extension, and was otherwise recorded as “multiple.”

The results of the proprioception test have three types of data: (1) continuous data refer to data that can be of any value, such as an error in distance or error in the degree of angle; (2) ordinal data refer to data with a set order or scale, such as the score of scales; and (3) categorical data refer to data reflecting types by classifying or grouping phenomena according to their properties; for instance, if a function is impaired or intact.

For motor function, all the tests involving the muscle, movement functions at the body function level under the framework of the International Classification of Functioning, Disability, and Health (ICF), the performance or independence of general functional tasks and self-care with upper limbs involved, and mobility according to the ICF and participation level were regarded as relevant measures to extract (23). The characteristics of the ICF domains of each motor function measure were extracted, including muscle tone, muscle strength, and movement function at the body function level, activity performance, independent activity level, and participation level. Besides, the result data type of tests for motor function, including continuous, ordinal, and categorical, was extracted to reflect the resolution, as that for the proprioception test (Table 2).

**Table 2:** Categories of proprioception and motor function measures of the included studies.

**Categories**	**Measures and studies**
1. Proprioception: subtypes	
Position sense	TLT (8, 9, 11, 15, 24, 25), JPS_shoulder (26), JPS_MCP (12, 27), JPS_trunk (28, 29), JPS_ankle (13, 30, 31), JPS_knee (13, 32), Stand-vision perturbed (10), Reach matching task (33), Arm push matching task (14), Target reaching task (22), Arm position test (24, 34), Hand position matching task (35)
Motion sense	Arm movement mirror-matching (11)
Position and motion sense	SIAS_Position_toe (36), rNSA-Proprioception (19, 37, 38), Elbow match task (21), FMA-UL_proprioception (20), Em-NSA_proprioception (8), JPS_ankle/knee/hip (39)
2. Proprioception: body parts	
Axial segment in weight-bearing conditions	Stand-vision perturbed (10), JPS_trunk (28, 29)
Upper limbs without weight-bearing	TLT (8, 9, 11, 15, 24, 25), JPS_MCP (12, 27), Arm push matching task (14), Arm movement mirror-matching (11), FMA-UL_proprioception (20), Elbow match task (21), Target reaching task (22), Arm position test (24, 34), Reach matching task (33), JPS_shoulder (26), Hand position matching task (35), Em-NSA_ proprioception (8), rNSA-Proprioception (37)
Lower limbs without weight-bearing	JPS_ankle/knee (13), rNSA-Proprioception (19, 38), JPS_Ankle (30, 31), SIAS_Position_toe (36), JPS_ankle, knee and hip (39)
3. Proprioception: matching side	
Contralateral matching	TLT (8, 9, 11, 15, 24, 25), Arm position test (24, 34), SIAS_Position_toe (36), rNSA-Proprioception (37, 38), Elbow match task (21), Arm movement mirror-matching (11), Hand position matching task (35), JPS_ankle/knee (13), FMA-UL_proprioception (20), Em-NSA_proprioception (8), JPS_1stMCP (12), JPS_ankle, knee and hip (39), JPS_ankle (31), JPS_knee (32)
Ipsilateral matching	Stand-vision perturbed (10), JPS_trunk (28, 29), Target reaching task (22), Arm push matching task (14), Reach matching task (33), JPS_MCP (27), JPS_shoulder (26), JPS_Ankle (30)
4. Proprioception: movement modes	
Passive-Active	TLT (8, 9, 11, 15, 24, 25), Arm position test (24, 34), SIAS_Position_toe (36), rNSA-Proprioception (37, 38), Elbow match task (21), Arm movement mirror-matching (11), Hand position matching task (35), JPS_ankle/knee (13), FMA-UL_proprioception (20), Em-NSA_proprioception (8), JPS_1stMCP (12), JPS_ankle, knee and hip (39), JPS_ankle (31), JPS_knee (32), Arm push matching task (14), Reach matching task (33), JPS_MCP (27)
Active–Active	Stand-vision perturbed (10), JPS_trunk (28, 29), Target reaching task (22)
Passive–Passive	JPS_shoulder (26), JPS_Ankle (30)
Passive–Perceptual	JPS_MCP (27)
5. Proprioception: number of joint planes	
Single	JPS_ankle/knee (13), rNSA-Proprioception (19, 38), JPS_Ankle (13, 30, 31), SIAS_Position_toe (36), JPS_ankle, knee and hip (39), JPS_knee (32), JPS_MCP (12, 27), JPS_shoulder (26), Elbow match task (21), FMA-UL_proprioception (20)
Multiple	TLT (8, 9, 11, 15, 24, 25), Stand-vision perturbed (10), Arm movement mirror-matching (11), Arm push matching task (14), Target reaching task (22), Arm position test (24, 34), Reach matching task (33), Hand position matching task (35), Em-NSA_proprioception (8), JPS_trunk (28, 29)
6. Proprioception: result data types	
Continuous	JPS_shoulder (26), JPS_trunk (28, 29), JPS_ankle (13, 30, 31), JPS_knee (13, 32), Stand-vision perturbed (10), Reach matching task (33), Arm push matching task (14), Target reaching task (22), Arm position test (24, 34), Hand position matching task (35), Arm movement mirror-matching (11), Elbow match task (21)
Ordinal	TLT (8, 9, 11, 15, 24), SIAS_Position_toe (36), rNSA-Proprioception (19, 37, 38), FMA-UL_proprioception (20), JPS_ankle, knee and hip (39)
Categorial	TLT (25), JPS_1stMCP (12)
7. Motor function: ICF domains	
Body function_muscle tone	MAS (12, 20, 24, 26, 34)
Body function_muscle strength	MVC (20), MI (8), Strength of grip (9), Strength of UE (12, 25) and LE (25)
Body function_movement	Reach-pick task&Pull-press task (21), Trunk movement tasks (28), FMA-LE (13, 29), FMA-UE (8, 9, 14, 19, 22, 26, 27, 37), Purdue Pegboard and Reaching task (24), Pointing task (32), AROM degree and joint individuation (12)
Activity_Performance	BBS (10, 29, 30), BBT (9, 27, 33), WMFT (33), ARAT (8, 9, 14), TUG (15, 38), FRT (15, 38), Gait speed (38), Stand_CoF sway velocity (38), Walking test (13), Postural function (25)
Activity_independence	BI (10, 36), FIM (11, 15, 24, 26), CMSA-arm and hand (24), WIS (38), Motor deficit (35), PASS (29)
Participation	MAL (9, 33), walk handicap (39), IADL (15)
Environment-specific activities performance	Fall incidence (38), Falls_faller (31)
8. Motor function: result data types	
Continuous	MVC (20), MI (8), Strength of grip(9), BBT (9, 27, 33), TUG (15, 38), Reach-pick task&Pull-press task (21), Pointing task (32), Trunk movement tasks (28), AROM degree&Joint individuation (12), FRT (15, 38), Gait speed (38), Stand_CoF sway velocity (38), Walking test (13), Fall incidence (38)
Ordinal	MAS (12, 20, 24, 26, 34), FMA-LE (13, 29), FMA-UE (8, 9, 14, 19, 22, 26, 27, 37), BBS (10, 29, 30), WMFT (33), ARAT (8, 9, 14), BI (10, 36), FIM (11, 15, 24, 26), CMSA-arm& hand (24), WIS (38), Motor deficit (35), PASS (29), MAL (9, 33), MAL (9, 33), walk handicap (39), IADL (15)
Categorial	Strength of UE and LE (25), Falls_faller (31), Postural function (25)

*ARAT, Action Research Arm Test; AROM, Active Range of Motion; BBS, Berg Balance Scale; BBT, Box and Block Test; BI, Barthel Index; CMSA, Chedoke-McMaster Stroke Assessment; CoP, center of pressure; Em-NSA, Erasmus-modified Nottingham sensory assessment; FIM, Functional Independence Measure; FMA, Fugl-Meyer Assessment, FRT, Functional Reach Test; JPS, joint position sense, IADL, Instrumental Activities of Daily Living Scale; LE, lower extremities; MAL, Motor Activity Log; MAS, Modified Ashworth Scale; ML, medial-lateral; MI, Motricity Index; UE, upper extremities; rNSA, revised Nottingham Sensory Assessment; SIAS, Stroke Impairment Assessment Set; TLT, thumb localizing test; TUG, Timed Up&Go; WMFT, Wolf Motor Function Test*.

A data extraction sheet was developed first, after which the work was independently performed by two researchers who further checked the accuracy of the extracted data.

### Quantitative Data Synthesis and Analysis

Comprehensive Meta-Analysis (CMA) software (version 3.0) was used for the meta-analysis. A CMA sheet was built, which consisted of study name, proprioception test name, motor function name, effect size data of correlation, and a series of moderators related to participant characteristics, and proprioception and motor function measures. The data value of the correlation between proprioception and motor function was standardized for meta-analysis according to the meaning of the test data value on corresponding functions. The correlation value was maintained when the paired proprioception and motor measures had both positive and negative indications for corresponding functions (Table 3). For example, the correlation between proprioception function measured by the Revised Nottingham Sensory Assessment (rNSA) of the wrist and motor function measured by Fugl-Meyer Assessment of the upper limb (FMA-UE) showed a coefficient of 0.419 in the study by Vlaar et al. (19). The values of rNSA and FMA-UE both have positive indications of function, with a higher value indicating better function. Therefore, a value of 0.419 was input in the CMA sheet as the effect size of the correlation. In another example of a correlation between proprioception function quantified by a spatial shift of the active hand from passive hands in the arm position test (larger shift and poorer function) and the motor function measured by the Modified Ashworth Scale (higher score and poorer function), the value of 0.096 in the study by Mochizuki et al. (34) was also input to the CMA sheet without change. On the contrary, the correlation value was changed to the opposite counterpart when the paired proprioception and motor measures had opposite indications for corresponding functions. In one instance of the correlation between proprioception function measured by rNSA (positive indication: higher score and better function) and motor function measured by Timed Up&Go (TUG) (negative indication: higher value and poorer function), the value of −0.12 in the study by Gorst et al. (34) was changed to 0.12, as the effect size in the CMA sheet.

**Table 3:** Characteristics of included studies.

**Studies**	**Participants**							**Proprioception test**	**Motor function measures**
	**Sample size**	**Age**	**Gender (F:M)**	**Time after onset (m)**	**Hemispheric lesion side (R: L)**	**Stroke type (Hemorrhage:Infarct)**	**Excluding Spatial neglect (Y/N)**		
Bonan et al. (10)	30	54.7 ± 10.6	9:21	1.9 ± 1.2	17:13		N	Stand-vision perturbed∧	1. BBS (0–56)
								-CoP sway (mm)-	2. BI (0–100)
Borstad and Nichols-Larsen (33)	12	64.2 ± 12.2	7:13	24.7 ± 24.7	7:5		Y	Reach matching task∧	1. BBT (no. of blocks)
								-error distance (cm)-	2. WMFT (no. of times)
									3. MAL
									-How much (0–5)
									-How well (0–5)
Cherpin et al. (14)	20	55.7 ± 11.0	8:12	13.2 ± 7.4	11:9		Y	Arm push matching task∧	1.FMA-UE (0–66)
								-Error distance (cm)-	2. ARAT (0–57)
								-Variability (cm)-	
Cho et al. (22)	10	54.6 ± 7.8	3:7	39.5 ± 46.2	1:9	3:7	Y	Target reaching task∧	FMA-UE (0–66)
								-Total error distance (cm)-	
								-Total movement distance (cm)-	
								-Average error distance (cm)-	
								-Number of click-	
								-Average movement distance (cm)-	
dos Santos et al. (26)	13	61.1 ± 10.6	–	45.6 ± 35.2	6:7		N	JPS_shoulder∧	1. FMA-UE (0–66)
								-Absolute error (degree)-	2. MAS (0–4)-
									3. FIM (18–126)
Dukelow et al. (24)	100	63 (21–90)	43:47	0.9 (0.2–2.7)	46:54		Y	1. TLT∧(0–3)-	1. MAS (0–4)-
								2. Arm position test∧	2. FIM (18–126)
								-Shift (cm)- -Trial variability (cm)-	4. Purdue Pegboard (pegs no.)
								-Area difference ratio-	5. CMSA-arm and hand (1–7)
									6. Reaching task
									-Reaction time (s)-
									-Error in direction (degree)-
									-Total time (s)-
									-Number of speed-peaks-
									-Postural preparation speed (cm/s)-
Fujita et al. (36)	108	73.1 ± 14.5	48:60	≈2 (1.7–2.3)	52:56	27:83	N	SIAS_Position_toe (0–3)Δ	BI-Walking (dependent/independent)
Gorst et al. (38)	163	67 ± 12	68:95	29 ± 46	77:75	37:115	N	rNSA-Proprioception (0–8)Δ	1. TUG(s)-
								-Distal	2. FRT (cm)
								-Proximal	3. Gait speed (m/s)
									4. WIS (12–60)-
									5. Stand: CoF sway velocity (mm/s)-
									6. Fall incidence (no.)-
Kantak et al. (21)	14	53 ± 15.4	5:9	78.9 ± 55.8	7:7		Y	Elbow match taskΔ	1. Reach-pick task
								-Error (degree)-	-Bimanual symmetrical time (s)-
									2. Pull-press task
									-Interval of asymmetric onset(s)-
									-Interval of asymmetric peak(s)-
									-Interval of asymmetric offset(s)-
Kenzie et al. (11)	146	60 ± 16	47:83	0.3 ± 0.2	75:67	0:146	N	1. TLT∧(0–3)-	1. FIM (18–126)
								2. Arm movement mirror-matching▴	
								-Response latency (ms)-	
								-Initial direction error-	
Leibowitz et al. (35)	22	62.1 (29–79)	12:10	2.5 (0.9–4.9)	11:11		Y	Hand position matching task∧	Motor deficits (0–3)-
								-Error (cm)-	
Liao et al. (28)	15	50.3 ± 7.7	8:7	55 ± 55	10:5	6:9	N	JPS_trunk∧	Trunk movement tasks
								-Error (degree)-	-Symmetry index of external abdominal oblique (0–0.5)-
									-Symmetry index of internal abdominal oblique (0–0.5)-
Lin (13)	21	65.2 ± 9.1	6:15	63.2 ± 55.5	13:8	9:12	N	JPS_ankle/knee∧	1. FMA-LE (0–34)
								-Error (degree)-	2. Walking test
									-Gait speed
									-Stride length
									-Step length (% body height)
									-Swing time
									-Support time
									-Double-leg-Stance-
Mercier et al. (20)	16	53.3 ± 13.2	7:9	59.1 ± 35.8	7:9		Y	FMA-UL_proprioception (0–2)^Δ^	1. MAS (0–4)-
									2. MVC (force:N)
									-Elbow flexion
									-Elbow extension
									-Shoulder flexion
									-Shoulder extension
Meyer et al. (8)	122	67 (58.8–76.1)	45:77	2.7 (0.3–6)	73:48	14:108	N	1.TLT (0–3)-∧	1. FMA-UE (0–66)
								2.Em-NSA_proprioception (0–8)^Δ^	2. ARAT (0–57)
									3. Ad-AHA Stroke (0–100)
									4. MI(0–100)
Mochizuki et al. (34)	70	60 (18–87)	21:49	10.5 (1–154)	36:34		N	Arm position test∧	MAS (0–4)-
								-Shift (cm)-	
								-Trial Variability (cm)-	
								-Area difference ratio-	
Niam et al. (30)	30	59.0 ± 13.8	12:18	10.9 ± 10.7	13:17		N	JPS_Ankle (0–1)∧	1. BBS (0–56)
									2. Stand sway (mm)-
									-In AP direction, with eye closed
									-In AP direction, with eye open
									-In ML direction, with eye open
Perry et al. (39)	147	55.5 ± 12.0	79:68	>3	79:68		N	JPS_ankle, knee and hip (0–3)^Δ^	Walking handicap (1–6)
Rand (9)	102	59.6 ± 29.8	33:69	20.9 ± 18.8	64:38		N	TLT (0–3)-∧	1. FMA-UE (0–66)
									2. ARAT (0–57)
									3. BBT (no. of blocks)
									4. Strength of grip (Kg)
									5. MAL
									-how much (0–5)
									-how well (0–5)
Rand (15)	64	59.9 ± 9.3	25:61	26.1 ± 18.3	23:41		N	TLT (0–3)-∧	1. FIM (18–126)
									2. TUG (time:s)-
									3. FRT (cm)
									4. IADL (0–8)
Ryerson et al. (29)	20	60.5(44–83)	9:11	63.6 ± 66	9:12		N	JPS_trunk∧	1. BBS (0–56)
								-Error (degree)-	2. FMA-LE (0–34)
									3. PASS (0–36)
Smith et al. (25)	216	≥60			131:87		N	TLT (0–1)∧	1. Strength (0–1)
									-UE
									-LE
									2. Postural function (0–1)
Soyuer and Ozturk (31)	100	62 ± 10.9	50:50	9 (6–18)	50:50	47:53	N	JPS_ankle∧	Falls_faller (0–3)
								-Error (degree)-	
Tsang et al. (32)	15	58.7 ± 7.5	6:9	90 ± 37	11:4		Y	JPS_knee∧	Pointing task
								-Error (degree)-	-Accuracy (mm)
Vlaar et al. (19)	30	64 ± 11	12:18	40 ± 47	17:13		N	rNSA-Proprioception_wrist (0–2)^Δ^	FMA-UE (0–66)
Wagner et al. (12)	46	64 ± 13	28:18	0.3 ± 0.1		10:30	N	JPS_1stMCP (0–1)∧	1. MAS (0–4)-
									2. AROM (degree)
									-Composite/
									-Shoulder
									-Elbow
									-Wrist
									3. Joint individuation (0–1)
									-Shoulder
									-Elbow
									-Wrist
									4. UE strength
									-Affected/unaffected ratio:0–1
Wu et al. (37)	147	53.4 ± 10.6	44:103	21.8 ± 18.3	75:72		Y	rNSA-Proprioception (0–2)^Δ^	FMA-UE (0–66)
Zbytniewska et al. (27)	30	64.5 ± 14.0	11:19	2.0 ± 1.1	19:11	21:9	N	1. JPS_2ndMCP∧	1. FMA-UE (0–66)
								-Error (degree)-	2. BBT (no. of blocks)
								2.2ndMCP_match task (slow/fast)^Δ^	
								-Error (degree)-	

*∧: Measures of position sense; Δ: measures of motion and position sense; ▴: measures of motion sense*.

*Measures: a minus sign following indicates the result data higher, the proprioception poorer; conversely without a minus sign following indicates higher result data and better proprioception*.

*AP, anterior posterior; ARAT, action research arm test; AROM, active range of motion; BBS, Berg Balance Scale; BBT, box and block test; BI, Barthel Index; CMSA, Chedoke-McMaster Stroke Assessment; CoP, center of pressure; Em-NSA, Erasmus-modified Nottingham sensory assessment; FIM, functional independence measure; FMA, Fugl-Meyer Assessment, FRT, Functional Reach Test; JPS, joint position sense, IADL, Instrumental Activities of Daily Living Scale; LE, lower extremities; MAL, Motor Activity Log; MAS, Modified Ashworth Scale; ML, medial-lateral; MI, Motricity Index; UE, upper extremities; rNSA, revised Nottingham Sensory Assessment; SIAS, Stroke Impairment Assessment Set; TLT, thumb localizing test; TUG, Timed Up and Go; WIS, Walking Impact Scale; WMFT, Wolf Motor Function Test*.

For studies with more than one measure of proprioception or motor function presented, a priority order of either proprioception or motor function, which was determined by their frequency presented across the included studies, was used to select the most common measure of proprioception or motor function for meta-analysis to minimize the heterogeneity across studies.

The correlation between proprioception and motor function in patients with stroke was analyzed. Statistical heterogeneity was assessed using the *I*^2^ statistic. If there was significant heterogeneity across studies, the random-effects model of the meta-analysis was used; otherwise, the fixed-effects model was used to analyze the correlation. For studies with significant heterogeneity, meta-regression tests or subgroup analyses were performed to explore the potential factors contributing to heterogeneity. Egger's test was used to evaluate the publication bias between the strength of the correlation and sample sizes. The strength of correlation was categorized into five levels according to the correlation coefficient (*r*): very weak (0.00–0.19), weak (0.20–0.39) , moderate (0.40–0.59), strong (0.60–0.79), and very strong (0.80–1.00) (40). The level of significance was set at *p* < 0.05, for all statistical tests.

## Results

### Study Identification and Selection

**Figure 1:**
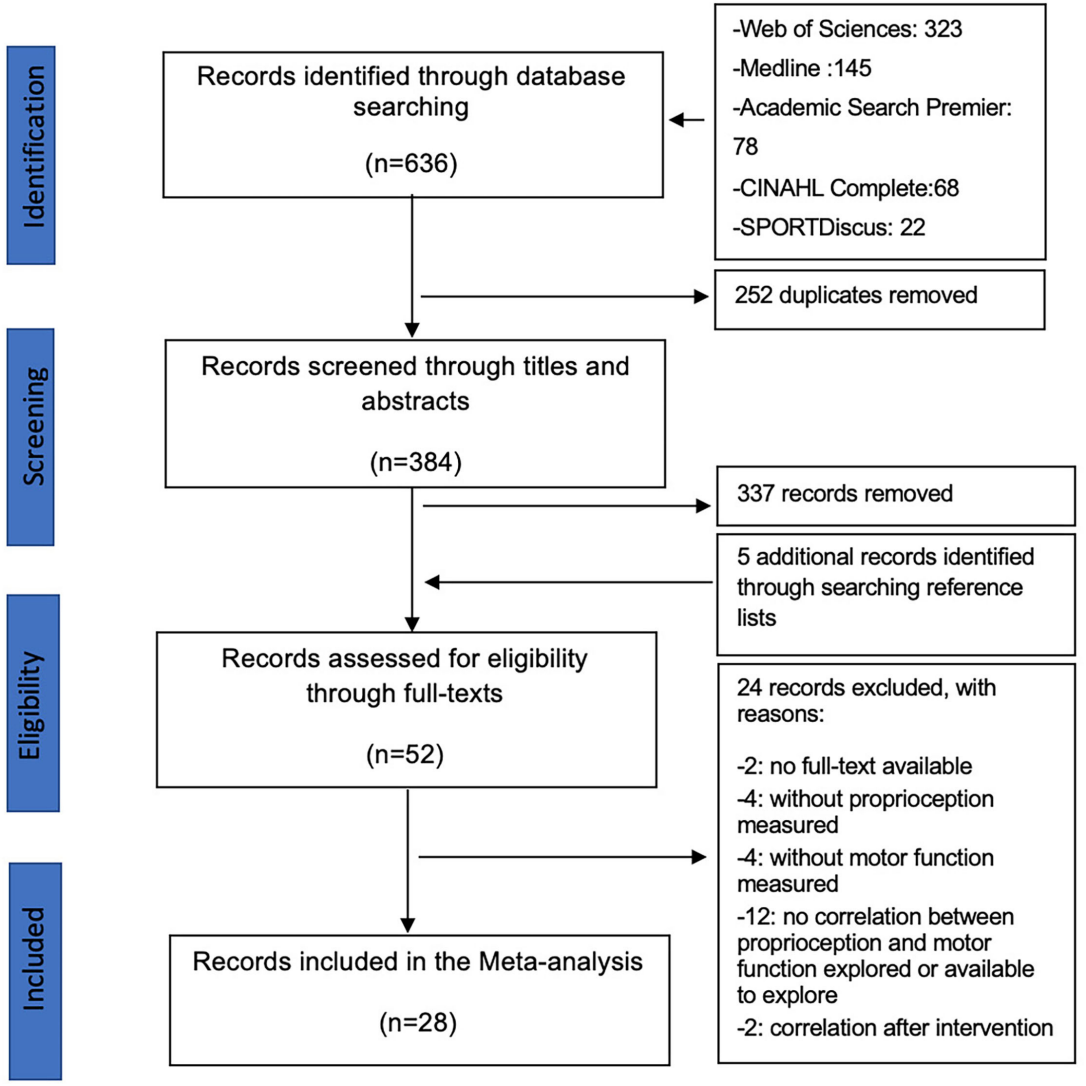
PRISMA diagram showing the trial flow.

A total of 636 articles were identified through database search; 384 articles passed the duplicate checking, and 47 articles passed the title-abstract screening. In addition to five articles identified by searching the reference lists for relevant articles, 52 articles were included in the eligibility assessment after reading the full text. A total of 28 articles fulfilled the selection criteria and were finally included in this review (Figure 1).

### Description of Included Studies

A total of 1,829 stroke participants with a mean age of >53 years were included in this review. The ratio of female-to-male patients was 0.69. The mean time since stroke ranged from 9 days (11, 12) to ~7 years (21). The ratio of persons with the right to left hemisphere stroke was 1.1. Among the 28 studies, nine (356 participants) excluded persons with spatial neglect using the subject selection criteria, while the other 19 (1,473 participants) did not mention spatial neglect in the selection criteria (Table 3).

For proprioception, the TLT and joint position sense (JPS) tests were the most frequently adopted measures in the reviewed studies. Other details of the proprioception tests are presented in Table 3. Position sense was tested in 22 studies (8–15, 22, 24–35, 37), while motion sense was examined in only one study (11), and combined position and motion senses were examined in eight studies (Tables 2, 3) (8, 19–21, 36–39). Upper limbs without weight-bearing were measured in 17 studies (8, 9, 11, 12, 14, 15, 20–22, 24–27, 33–35, 37), followed by lower limbs without weight-bearing in eight studies (13, 19, 30–32, 36, 38, 39), and the axial segment in weight-bearing conditions in three studies (10, 28, 29). The contralateral matching was adopted in 20 studies, where persons with stroke were asked to use the unaffected side to match the pre-set position or motion of the affected side (8, 9, 11–13, 15, 19–21, 24, 25, 29, 31, 32, 34–39). Other eight studies used ipsilateral matching task in which the affected side or axial segment was only involved (10, 14, 22, 26–28, 30, 33). The movement mode of “passive-then-active” was the most frequently adopted in 22 studies (8, 9, 11–15, 19–21, 24, 25, 29, 31–39), followed by “active-then-active” in 3 studies (10, 22, 28), “passive-then-passive” in 2 studies (26, 30), and “passive-then-perceptual ” in one study (27).

Half of the studies measured proprioception within a single joint plane, such as using the JPS test (8, 12, 13, 19–21, 26, 30–32, 36, 38, 39), while the other half assessed proprioception of multiple joint plane using methods, such as the TLT (9–11, 14, 15, 22, 24, 25, 28, 29, 33–35). Half of the studies used proprioception instruments with continuous data in the results (10, 13, 14, 21, 22, 26–35). The other half adopted clinical tests with ordinal or categorical data in the results, such as the TLT and proprioception part of the rNSA (Tables 2, 3) (8, 9, 11, 12, 15, 19, 20, 24, 25, 36–39).

For motor function, the FMA-UE section and MAS were the most common measures used in the reviewed studies. Other details of the motor function tests are presented in Table 3. Movement function was measured in most of the studies (12 studies), followed by activity performance measured in five studies, muscle tone function and activity independence both examined in four studies, and muscle strength, environment-specific activity performance, and participation measured in only one study. Most tests for motor function have ordinal data in the results (8–15, 19, 20, 22, 24, 26, 27, 29, 30, 34–37, 39).

### Methodological Quality

All the included studies were evaluated based on the modified QUADAS-2, the results of which are shown in Table 4. None of them scored “YES” for all 11 items. For the quality of patient selection, none of them recruited subjects based on a consecutive series or a random sample, and most of them did not mention their sampling information. None of the studies adopted a case-control design or mentioned the related information. None of the studies had inappropriate exclusions, such as those with proprioceptive impairment or motor deficit, or mentioned the relevant information. Regarding the quality of the proprioception tests used, one study assessed the proprioceptive function 1 week after motor function (26), while five studies (11, 14, 24, 27, 33), tested the proprioception and motor function at one time node. Others were deemed unclear because of insufficient information about the interval of the tests. In addition, most of the studies adopted proprioception tests with a standard scoring scale or pre-specified threshold, except for two studies where a self-defined threshold for an innovative measure or non-standard scoring method for a common test were mentioned only in the “Results” section (14, 25). Regarding the quality of the motor function test used, in addition to the same situation with test intervals as the proprioception tests, all of the studies assessed motor function correctly. Regarding the flow and timing, only one study measuring proprioceptive and motor function at two time nodes could have a bias, influencing the later measures (26). Others either conducted measures at one time node or did not report the information. All the participants underwent motor function tests in all but three of the studies (15, 25, 38), where some participants did not because of the study design (15) or incomplete measures (25, 38). The motor function tests were the same for all the participants within each study. All the participants who completed measures were included in the data analysis in all but three of the studies where only participants with proprioceptive impairment were included (26), or the data with the technical error were excluded from the analysis (30).

**Table 4:** QUADAS-2 assessments of included studies.

**Studies**	**Patient selection**			**Index test**		**Reference standard**		**Flow and timing**			
	**Without bias in sampling**	**Without bias in design**	**Without bias in selection criteria**	**Without bias from knowing reference test results**	**With pre-specified threshold**	**Correctly reflecting motor function**	**Without bias from knowing index test results**	**With proper interval between two tests**	**All subjects receiving reference tests**	**The same Reference tests for all subjects**	**All subjects in data analysis**
Bonan et al. (10)	N	Y	Y	Unclear	Y	Y	Unclear	Unclear	Y	Y	Y
Borstad and Nichols-Larsen (33)	N	Y	Y	Y	Y	Y	Y	Y	Y	Y	Y
Cherpin et al. (14)	N	Y	Y	Y	N	Y	Y	Y	Y	Y	Y
Cho et al. (22)	N	Y	Y	Unclear	Y	Y	Unclear	Unclear	Y	Y	Y
dos Santos et al. (26)	N	Y	Y	N	Y	Y	Y	N	Y	Y	N
Dukelow et al. (24)	N	Y	Y	Y	Y	Y	Y	Y	Y	Y	Y
Fujita et al. (36)	N	Y	Y	Unclear	Y	Y	Unclear	Unclear	Y	Y	Y
Gorst et al. (38)	N	Y	Y	Unclear	Y	Y	Unclear	Unclear	N	Y	Y
Kantak et al. (21)	Unclear	Unclear	Unclear	Unclear	Y	Y	Unclear	Unclear	Y	Y	Y
Kenzie et al. (11)	N	Y	Y	Y	Y	Y	Y	Y	Y	Y	Y
Leibowitz et al. (35)	N	Unclear	Unclear	Unclear	Y	Y	Unclear	Unclear	Y	Y	Y
Liao et al. (28)	Unclear	Y	Y	Unclear	Y	Y	Unclear	Unclear	Y	Y	Y
Lin (13)	N	Y	Y	Unclear	Y	Y	Unclear	Unclear	Y	Y	Y
Mercier et al. (20)	Unclear	Y	Y	Unclear	Y	Y	Unclear	Unclear	Y	Y	Y
Meyer et al. (8)	Unclear	Y	Y	Unclear	Y	Y	Unclear	Unclear	Y	Y	Y
Mochizuki et al. (34)	Unclear	Y	Y	Unclear	Y	Y	Unclear	Unclear	Y	Y	Y
Niam et al. (30)	N	Y	Y	Unclear	Y	Y	Unclear	Unclear	Y	Y	N
Perry et al. (39)	Unclear	Y	Y	Unclear	Y	Y	Unclear	Unclear	Y	Y	Y
Rand (9)	N	Y	Y	Unclear	Y	Y	Unclear	Unclear	Y	Y	Y
Rand (15)	N	Y	Y	Unclear	Y	Y	Unclear	Unclear	N	Y	N
Ryerson et al. (29)	N	Y	Y	Unclear	Y	Y	Unclear	Unclear	Y	Y	Y
Smith et al. (25)	N	Y	Y	Unclear	N	Y	Unclear	Unclear	N	Y	Y
Soyuer and Ozturk (31)	Unclear	Y	Y	Unclear	Y	Y	Unclear	Unclear	Y	Y	Y
Tsang et al. (32)	N	Y	Y	Unclear	Y	Y	Unclear	Unclear	Y	Y	Y
Vlaar et al. (19)	Unclear	Y	Y	Unclear	Y	Y	Unclear	Unclear	Y	Y	Y
Wagner et al. (12)	N	Y	Y	Unclear	Y	Y	Unclear	Unclear	Y	Y	Y
Wu et al. (37)	Unclear	Y	Y	Unclear	Y	Y	Unclear	Unclear	Y	Y	Y
Zbytniewska et al. (27)	N	Y	Y	Y	Y	Y	Y	Y	Y		

### Meta-Analysis Results

**Figure 2:**
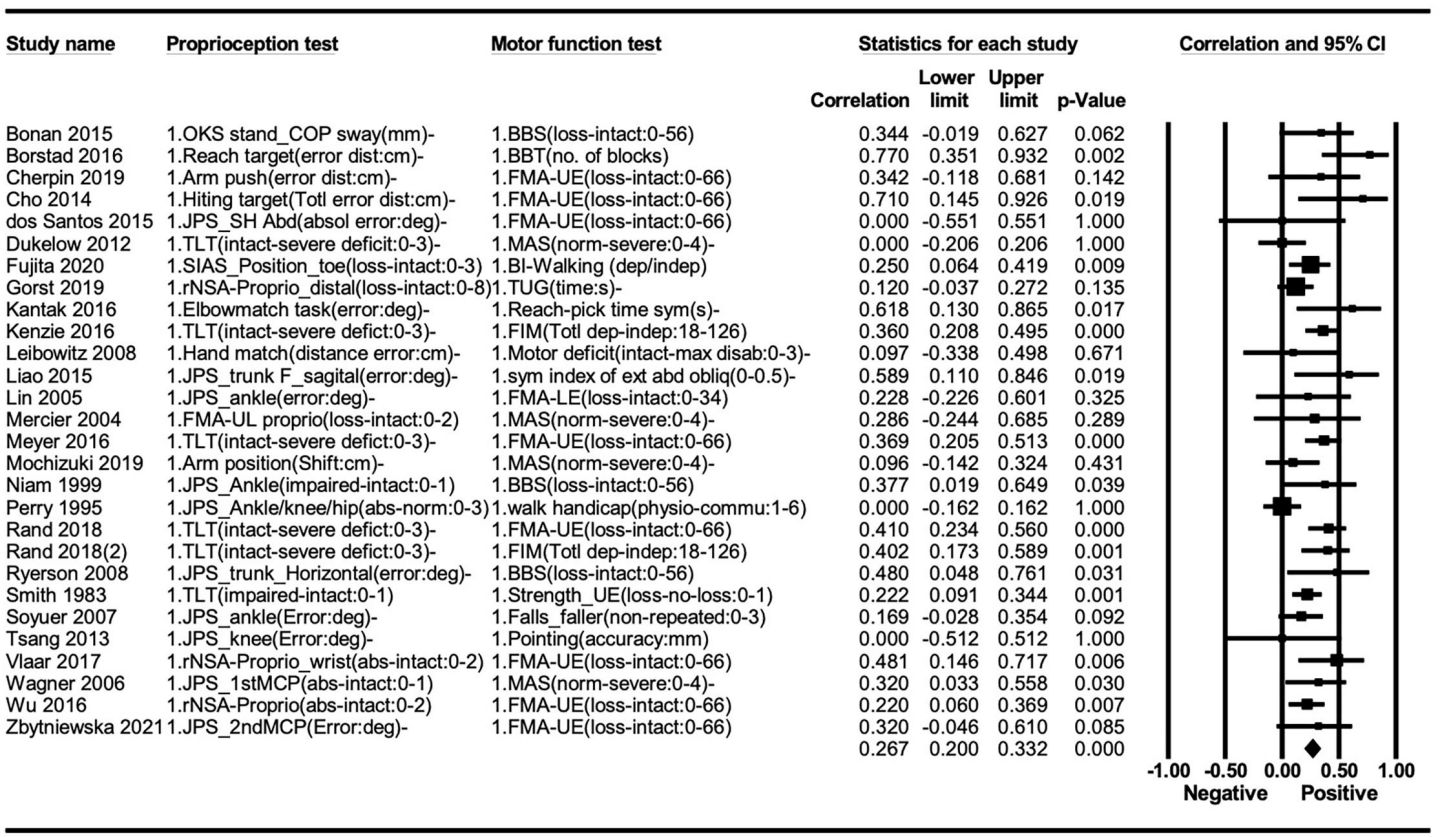
Meta-analysis: Association between proprioception and motor function after stroke. Random-effect model of analysis: I^2^ = 45* across all studies (*p < 0.05).

**Figure 3:**
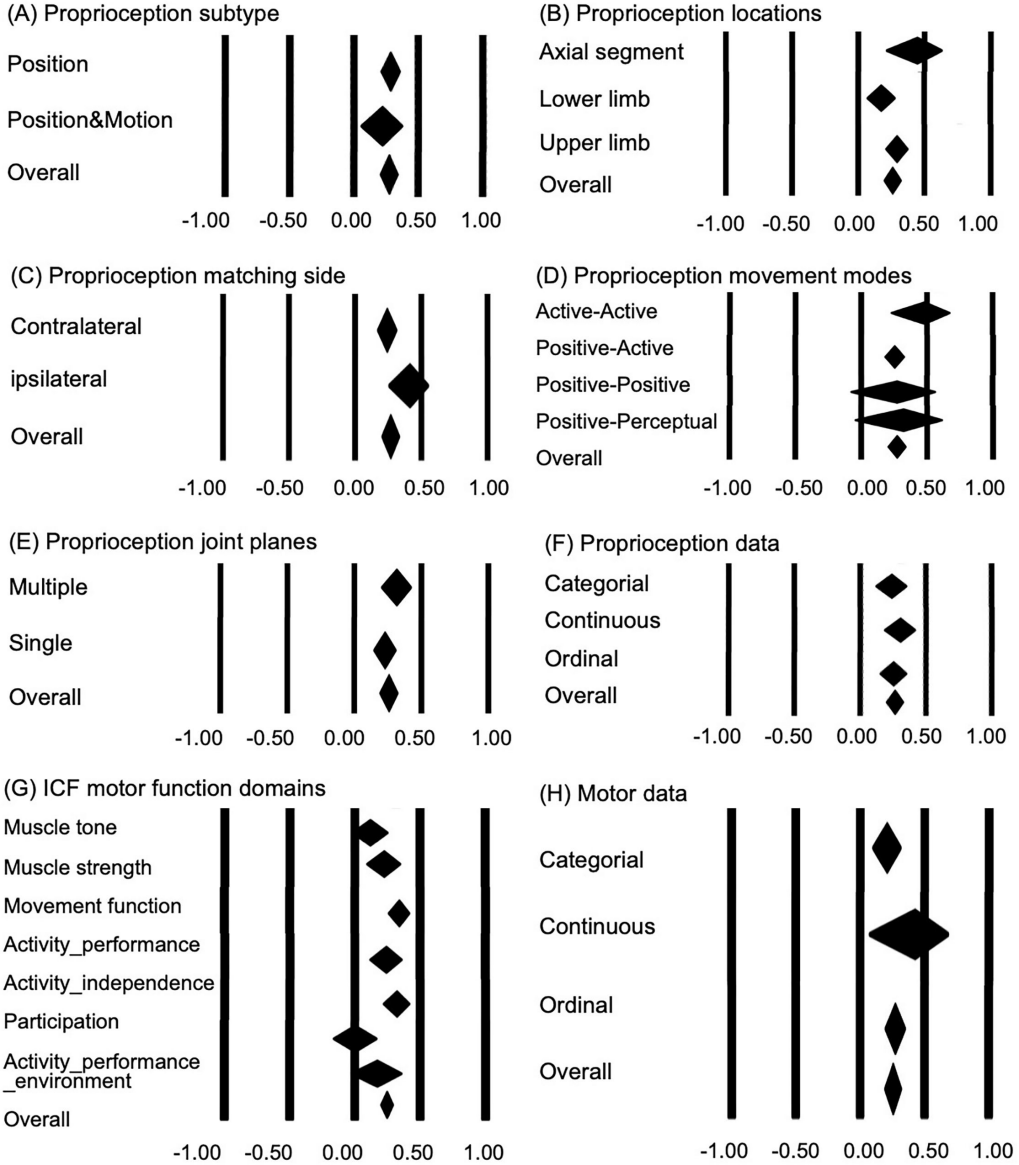
Subgroup analysis: association of proprioception with motor function after stroke. **(A)** Difference between proprioception subtypes measured in the tests (between-group difference: *p* = 0.456). **(B)** Influence of body parts involved in the proprioception tests (between-group difference: *p* = 0.055). **(C)** Influence of matching side involved in the proprioception tests (side involved) (between-group difference: *p* = 0.050). **(D)** Influence of the movement modes involved in the proprioception tests (between-group difference: *p* = 0.380). **(E)** Influence of joint planes measured in the proprioception tests (between-group difference: *p* = 0.205). **(F)** Influence of result acuity of the proprioception tests (between-group difference: *p* = 0.710). **(G)** Influence of ICF motor function domains (between-group difference: *p* = 0.003). **(H)** Influence of result acuity of the motor function tests (between-group difference: *p* = 0.364).

The correlation between proprioception and motor function was significant (*r* = 0.267, *p* < 0.05); however, there was significant heterogeneity across the studies (*I*^2^ = 45%, *p* < 0.05) (Figure 2). For subtypes of proprioception, the function of position sense only (*r* = 0.284, *p* < 0.05) and combined position and motion sense (*r* = 0.219, *p* < 0.05) showed a comparable correlation with motor function (between-group difference: *p* > 0.05) (Figure 3A). For proprioception at different locations, near-significant differences in correlation with motor function (between-group difference: *p* = 0.055) occurred among the axial segment in weight-bearing conditions (*r* = 0.443, *p* < 0.05), upper limb (*r* = 0.292, *p* < 0.05), and lower limb without weight-bearing (*r* = 0.174, *p* < 0.05) (Figure 3B). Proprioception measured with ipsilateral matching (overall *r* = 0.412) showed a near-to-significantly stronger correlation with motor function than contralateral matching (*r* = 0.240) (between-group difference: *p* = 0.050) (Figure 3C). Proprioception measured with different movement modes showed no significant difference in correlation with motor function (between-group difference: *p* > 0.05) (Figure 3D). The proprioception sense in single and multiple movement planes showed comparable relationships with motor function (between-group difference: *p* > 0.05) (Figure 3E). Comparing the influence of the resolution of proprioception measures on the correlation with motor function, no significant difference (between-group difference: *p* > 0.05) was found among the proprioception with continuous data, ordinal data, and categorical data results (Figure 3F).

The domains of motor function showed significant differences in correlation with proprioception (between-group difference, *p* < 0.05). Muscle strength (*r* = 0.222, *p* < 0.05), movement function (*r* = 0.338, *p* < 0.05), activity performance (*r* = 0.239, *p* < 0.05), and activity independence (*r* = 0.319, *p* < 0.05) were significantly correlated with proprioception function, while other domains showed no significant correlation with proprioception function (*p* > 0.05) (Figure 3G). The resolution of motor function measures demonstrated no significant influence on the correlation between proprioception and motor function (between-group difference: *p* > 0.05) (Figure 3H). Please see the Appendix I in Supplementary Material for full edition of Figure 3.

Upon further exploring the factors contributing to the heterogeneity of correlation between proprioception and motor function based on the characteristics of participants, including age, sex ratio, time after stroke onset, the ratio of persons with a right to those with left hemisphere stroke, and excluding persons with spatial neglect, we found that none of them showed a significant contribution to the strength of correlation (*p* > 0.05).

There was a publication bias between the strength of the correlation and the sample sizes found by Egger's test (*p* < 0.05).

## Discussion

This study first investigated the correlation between proprioception and motor function by considering their characteristics in a meta-analysis. The findings of this study verify our hypothesis that a significant but weak correlation (*r* = 0.267) exists between proprioception and motor function in patients with stroke (40). The weak correlation could result from the formal difference in movements involved in proprioception tests and motor function tests, and the poor ecological validity of proprioception tests that the testing conditions are different from normal functions in daily activities (41). Although generally weak, the correlations demonstrated moderate heterogeneity across studies (*r* = 0–0.770, *I*^2^ = 45%). Based on the heterogeneity, we identified several factors contributing to the correlation strength from the characteristics of proprioception and motor function tests.

### Clinical and Research Implications

#### Characteristics of Proprioception Tests and Factors Influencing the Correlation Between Proprioception and Motor Function

Among subtypes of proprioception, position sense has been the most commonly investigated over other senses when exploring the association with motor function in previous studies. During tests for position sense, participants were asked to localize the position of their test thumb or to match a reference joint/target position. An error of localized or reproduced position was recorded to reflect the accuracy of position sense. Only one study examined motion sense (11), where participants were required to mirror a reference movement using the contralateral limb, and the latency or trajectory error of mirrored movement was recorded to quantify the motion sense. Several studies adopted movement matching test but reported the result with position and motion sense integrated together, which we categorized as a subtype of combined position and motion sense (19–21, 36–39).

Most tests for position sense, such as JPS and various matching tests, used contralateral matching tasks with “passive-then-active” movement mode in previous studies. This test manner has been argued with lower testing validity than tests for motion sense due to incomparable movement mode and proprioceptive information available during reference position generation and replication (41). The TLT, another commonly used test for position sense, measures the position of the thumb in space, is regarded with less content validity for proprioception than the JPS, and tests for motion sense (42). Therefore, we questioned that whether position sense has a weaker correlation with motion function than motion sense. In a unique study examining both the motion sense and position sense, the correlation with motor function showed comparable significance between the two senses (*r* = 0.360 for position sense measured by TLT, *r* = 0.325 and 0.397 for motion sense quantified with the reaction latency and direction error of movement mirroring test, respectively) (11). Our meta-analysis study found a comparable association with motion function between position sense and the combined position and motion sense. These findings may indicate that position sense and motion sense could be similarly important for motor function recovery after stroke. However, some caution must be taken to interpret the above-mentioned findings as poor ecological validity for most proprioception tests could make their difference less obvious.

Most proprioception tests targeted limbs in non-weight-bearing conditions in the included studies. This approach is supposed to ensure the purity of proprioception input; however, it weakens the ecological validity, especially for the lower limb, since most daily functional activities are performed in weight-bearing conditions (41). This notion is verified by our finding that proprioception in the lower limb without weight-bearing only showed a very weak association with motor function after stroke (*r* = 0.174), much less than that in the axial segment with the trunk and lower limb involved in weight-bearing conditions (*r* = 0.443). We further found that similarly in non-weight-bearing conditions, proprioception in the upper limb showed a higher association with motor function (*r* = 0.292) than that in the lower limb. It is possibly because movements at the upper limb are usually executed without weight-bearing, in other words, proprioception tests for the upper limbs without weight-bearing have higher ecological validity than those for the lower limbs. However, for the phenomenon of weaker correlation for proprioception tested in the upper limbs without weight-bearing than that tested in the axial segment with weight-bearing, ecological validity related to weight-bearing is improper to explain, but other potential factors could exist, such as matching manner, which will be discussed in later paragraphs. Based on these findings, weight-bearing could be proposed as an important component of proprioception training for the lower limb or axial segment to boost the effect on improving motor function.

Contralateral matching tasks were adopted for testing proprioception more frequently than ipsilateral matching tasks in the included studies. The use of contralateral matching tasks can make the movements of reference generation and replication nearly synchronous, thereby largely reducing the need for memory-based matching. In spite of this advantage, based on the neural mechanism underlying the role of proprioception on motor control (2, 3), it is almost assured that contralateral matching requires more inter-hemispheric communication than ipsilateral matching. This disadvantage leads to a larger matching error using contralateral matching than ipsilateral matching, which has been verified in healthy subjects (43). For patients with impaired inter-hemispheric communication after stroke, the contralateral matching tasks should be greatly challenging, thereby could produce a much larger error than ipsilateral matching. Moreover, we hypothesized that the proprioception measured using the contralateral matching task has a weaker correlation with motor function than using an ipsilateral matching task. The hypothesis has been affirmed by our finding that proprioception measured ipsilateral matching tasks (*r* = 0.412) moderately correlated with motor function, more strongly than proprioception measured using contralateral matching tasks (*r* = 0.240) (40). This finding indicates a higher priority of ipsilateral matching tasks in proprioception measurement and at the initial stages of proprioceptive movement training.

For the reference generation and replication movement during matching tasks, different movement modes, such as passive-then-active, are suggested reducing the testing validity (41). The sensitivity of muscle spindles during active movement is increased *via* the gamma motor system (43, 44). Different modes of movement excite different types and number of proprioceptive inputs, which would generate system error of matching task. Based on the advantage of active movement, we speculate that the “active-then-active” is the most valid manner for testing proprioception and have a stronger correlation with motor function than other modes. The speculation could be partially supported by our results. In our study, the active-then-active mode of movement showed a much higher correlation with motor function (*r* = 0.479) than other modes, despite without a between-group difference in subgroup analysis which might be possibly due to a small number of studies adopting this mode. Besides, the test with multi-plane movement demonstrated a slightly stronger association with motor function (*r* = 0.315) than that with single-plane (*r* = 0.228), despite without a between-group difference in subgroup analysis. This finding could be explained by the ecological validity of the test since daily movements almost occur in multi-planes with multi-joints. Moreover, in most of the included studies, motor function was measured by a composite test or global performance with multi-joints involved, such as FMA and Berg Balance Scale (BBS), thus had higher formal similarity with multi-planes movement in proprioception tests, which could be another reason of the above finding. Thus, in the movement mode, a joint plane could be necessary to consider in designing proprioceptive training, giving priority to active mode, multi-planes, and multi-joints.

Nowadays, various technology-assisted proprioception evaluation systems have been developed for enhancing the precision of proprioception tests. Usually, these kinds of systems are used for biofeedback-augmented proprioceptive training. Despite the advanced functions and uses of the technology-assisted system, it cannot replace the clinical proprioception tests. Most clinical proprioception tests use ordinal or categorical data to record results that have less resolution (8, 9, 11, 12, 15, 19, 20, 24, 25, 27, 31, 32, 37) but have been found sensitive enough to detect impairments of proprioception (43). We found proprioception presented with different types of data showed no difference in the correlation with motor function, which further verifies the validity of clinical proprioception tests.

#### Factors of Motor Function With a Contribution to the Correlation Between Proprioception and Motor Function

This study included all measures related to motor function across ICF domains. We found that movement function (*r* = 0.338), activity performance (*r* = 0.239), and independence (*r* = 0.319) were more strongly correlated with proprioception than with muscle strength (*r* = 0.222), muscle tone (*r* = 0.116), environment-specific activity performance (*r* = 0.169), and participation (*r* = 0.000). These findings match the physiological mechanisms of motor control (45) and ICF concepts (46). At the body function level, movement function requires proprioception feedforward for motor planning and feedback for motor adaptation, which processes within complex brain networks (2, 11), while muscle tone relies on proprioception input to regulate through the stretch reflex, which works at and below the spinal cord level (45). Hemisphere stroke impairs the central, but not the peripheral-pathway of proprioception, which makes proprioception function more correlated with centrally controlled movement function than with relatively peripherally regulated muscle tone function in persons with stroke. Muscle strength, in spite of only one study included for analysis, showed a significant but weaker correlation with proprioception function than with movement function. The mechanism underlying this correlation could be different. Muscle strength is thought to be independent of proprioception, based on the evidence that pure sensory stroke impairs movement coordination but not muscle strength (47). However, centrally induced muscle weakness is usually accompanied by sensory disturbance after common cerebral artery strokes. Their correlation mainly exists due to homogeneity or similarity to some degree in neural lesions and repair, which is weaker than the direct influence of proprioception on movement function. Furthermore, within the ICF framework, the activity domain links directly with the body function domain where the proprioception function belongs (46), which could explain the stronger correlation of proprioception with activity than with environment-specific activity performance (number of falls) and participation. This finding indicates that proprioception-augmented training could be more effective in improving the movement function, activity performance, and independence than improving the muscle strength, muscle tone function, and environment-specific activity performance and participation.

Besides, the motor function measured with continuous data for the result showed a much stronger association with proprioception (*r* = 0.424) than those with ordinal and categorical data (*r* = 0.272 and 0.205, respectively), in spite of no significant between-group difference. The phenomenon implies that motor function measures with higher resolution might be more sensitive to detect the effect of proprioception-augmented movement training.

#### Other Potential Factors Influencing the Correlation Between Proprioception and Motor Function

Furthermore, to our knowledge, some characteristics of the disease could affect proprioception function, such as duration after stroke onset (25), side of the hemispheric lesion (11, 21, 48), and spatial neglect (8). We further explored the contributing roles of the aforementioned disease features and the demographic features of participants on the heterogeneity of the correlation between proprioception and motor function, with no significant contribution found. This finding indicates that regardless of stroke status and when or how a stroke occurs, proprioception is important for motor function after stroke.

### Limitations

Despite its significant findings, this study had several limitations. First, the assessment of methodological quality revealed potential sources of bias in the reviewed studies. The participants in most of the studies were recruited by convenience sampling, which could make them less representative. Fortunately, none of the characteristics of participants or their disease significantly contributed to heterogeneity, which could minimize the influence of potential bias. Moreover, the time between the proprioception test and motor function measurements in most of the studies has not been clearly described. However, it is most likely that they were assessed in one session without an obvious interval in cross-sectional studies, which has a low bias in test conduction and result in interpretation without knowing the results of the other test in advance. Second, only one study measured motion sense, and no study measured force sense in exploring the association with motor function (11). Regarding the main subtypes of proprioception, it is suggested that motion and force sense be measured routinely as position sense for a more in-depth investigation of proprioception. Third, publication bias between the strength of the correlation and the sample size was significant. Removing the seven studies with a sample size of <20 (20–22, 26, 28, 32, 33), the findings were similar to those previously reported (Appendices II, III in Supplementary Material), which affirm the representativeness of those studies, albeit with a small size. Fourth, only articles published in English may not represent all evidence. Fifth, based on the selection priority order of measures, less commonly used measures had a much lower possibility of being included in the meta-analysis than the more commonly used measures, which could reduce the power of sub-group analysis. Therefore, caution should be exercised when interpreting the results.

## Conclusion

In conclusion, this meta-analysis confirmed that proprioceptive impairment correlates with motor dysfunction in patients with stroke. The subgroup analysis has identified several factors with a positive contribution to the correlation between proprioception and motor function. They are proprioception tested in the axial segment with weight-bearing, proprioception measured with an ipsilateral matching task, and motor function in ICF domains of movement function, activity performance, and activity independence. These findings might be useful in directing the design of optimal proprioception enhancing movement training, but must be interpreted with some degree of caution since the correlation does not imply causation and might be underestimated by attributes of current tests for proprioception and motor function. Further studies are needed to clarify the cause-effect relationship.

## Data Availability Statement

The original contributions presented in the study are included in the article/Supplementary Material, further inquiries can be directed to the corresponding authors.

## Author Contributions

XS contributed to the conception and design of the study and provided guidance during the whole process of the study. YY, YC, and TL contributed to study searching and screening, quality assessment, data extraction, and data analysis. YY wrote the first draft of the manuscript, and the other authors reviewed the manuscript. All authors approved the final version of the paper.

## Funding

This study was supported by the National Key R&D Program of China (Project code: 2020YFC2004202). The findings and conclusions in this study are those of the authors and do not necessarily represent the official position of the National Key R&D Program of China.

## Conflict of Interest

The authors declare that the research was conducted in the absence of any commercial or financial relationships that could be construed as a potential conflict of interest.

## Publisher's Note

All claims expressed in this article are solely those of the authors and do not necessarily represent those of their affiliated organizations, or those of the publisher, the editors and the reviewers. Any product that may be evaluated in this article, or claim that may be made by its manufacturer, is not guaranteed or endorsed by the publisher.
