# Supplementary material for: Correlation Between Proprioceptive Impairment and Motor Deficits After Stroke: A Meta-Analysis Review
Source: Front Neurol. 2022 Jan 13;12:688616. doi: 10.3389/fneur.2021.688616 (PMC8793362; doi:10.3389/fneur.2021.688616)
Supplement: Supplementary file 1 [file Data_Sheet_1.pdf]

Group by	Study name	Proprioception test	Motor function test	Statistics for each study			
1.Proprio subtype				Correlation	Lower limit	Upper limit	p-Value
position	Bonan 2015	1.OKS stand_COP sway(mm)-	1.BBS(loss-intact:0-56)	0.344	-0.019	0.627	0.062
position	Borstad 2016	1.Reach target(error dist:cm)-	1.BBT(no. of blocks)	0.770	0.351	0.932	0.002
position	Cherpin 2019	1.Arm push(error dist:cm)-	1.FMA-UE(loss-intact:0-66)	0.342	-0.118	0.681	0.142
position	Cho 2014	1.Hiting target(Totl error dist:cm)-	1.FMA-UE(loss-intact:0-66)	0.710	0.145	0.926	0.019
position	dos Santos 2015	1.JPS_SH Abd(absol error:deg)-	1.FMA-UE(loss-intact:0-66)	0.000	-0.551	0.551	1.000
position	Dukelow 2012	1.TLT(intact-severe deficit:0-3)-	1.MAS(norm-severe:0-4)-	0.000	-0.206	0.206	1.000
position	Kenzie 2016	1.TLT(intact-severe defict:0-3)-	1.FIM(Totl dep-indep:18-126)	0.360	0.208	0.495	0.000
position	Leibowitz 2008	1.Hand match(distance error:cm)-	1.Motor deficit(intact-max disab:0-3)-	0.097	-0.338	0.498	0.671
position	Liao 2015	1.JPS_trunk F_sagital(error:deg)-	1.sym index of ext abd obliq(0-0.5)-	0.589	0.110	0.846	0.019
position	Lin 2005	1.JPS_ankle(error:deg)-	1.FMA-LE(loss-intact:0-34)	0.228	-0.226	0.601	0.325
position	Meyer 2016	1.TLT(intact-severe defict:0-3)-	1.FMA-UE(loss-intact:0-66)	0.369	0.205	0.513	0.000
position	Mochizuki 2019	1.Arm position(Shift:cm)-	1.MAS(norm-severe:0-4)-	0.096	-0.142	0.324	0.431
position	Niam 1999	1.JPS_Ankle(impaired-intact:0-1)	1.BBS(loss-intact:0-56)	0.377	0.019	0.649	0.039
position	Rand 2018	1.TLT(intact-severe defict:0-3)-	1.FMA-UE(loss-intact:0-66)	0.410	0.234	0.560	0.000
position	Rand 2018(2)	1.TLT(intact-severe defict:0-3)-	1.FIM(Totl dep-indep:18-126)	0.402	0.173	0.589	0.001
position	Ryerson 2008	1.JPS_trunk_Horizontal(error:deg)-	1.BBS(loss-intact:0-56)	0.480	0.048	0.761	0.031
position	Smith 1983	1.TLT(impaired-intact:0-1)	1.Strength_UE(loss-no-loss:0-1)	0.222	0.091	0.344	0.001
position	Soyuer 2007	1.JPS_ankle(Error:deg)-	1.Falls_faller(non-repeated:0-3)	0.169	-0.028	0.354	0.092
position	Tsang 2013	1.JPS_knee(Error:deg)-	1.Pointing(accuracy:mm)	0.000	-0.512	0.512	1.000
position	Wagner 2006	1.JPS_1stMCP(abs-intact:0-1)	1.MAS(norm-severe:0-4)-	0.320	0.033	0.558	0.030
position	Wu 2016	1.rNSA-Proprio(abs-intact:0-2)	1.FMA-UE(loss-intact:0-66)	0.220	0.060	0.369	0.007
position	Zbytniewska 2021	1.JPS_2ndMCP(Error:deg)-	1.FMA-UE(loss-intact:0-66)	0.320	-0.046	0.610	0.085
position				0.284	0.214	0.351	0.000
Position&motion	Fujita 2020	1.SIAS_Position_toe(loss-intact:0-3)	1.BI-Walking (dep/indep)	0.250	0.064	0.419	0.009
Position&motion	Gorst 2019	1.rNSA-Proprio_distal(loss-intact:0-8)	1.TUG(time:s)-	0.120	-0.037	0.272	0.135
Position&motion	Kantak 2016	1.Elbowmatch task(error:deg)-	1.Reach-pick time sym(s)-	0.618	0.130	0.865	0.017
Position&motion	Mercier 2004	1.FMA-UL proprio(loss-intact:0-2)	1.MAS(norm-severe:0-4)-	0.286	-0.244	0.685	0.289
Position&motion	Perry 1995	1.JPS_Ankle/knee/hip(abs-norm:0-3)	1.walk handicap(physio-commu:1-6)	0.000	-0.162	0.162	1.000
Position&motion	Vlaar 2017	1.rNSA-Proprio_wrist(abs-intact:0-2)	1.FMA-UE(loss-intact:0-66)	0.481	0.146	0.717	0.006
Position&motion				0.219	0.056	0.371	0.009
Overall				0.273	0.209	0.335	0.000

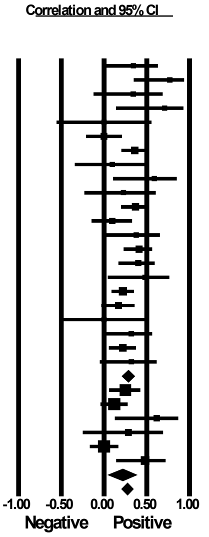

(A) Random-effect model of analysis: I^2=33%, 59%* across studies measuring position sense, combined position and motion senses respectively. (*:p<0.05)

Group by	Study name	Proprioception test	Motor function test	Statistics for each study			
2. Proprio local				Correlation	Lower limit	Upper limit	p-Value
axial seg	Bonan 2015	1.OKS stand_COP sway(mm)-	1.BBS(loss-intact:0-56)	0.344	-0.019	0.627	0.062
axial seg	Liao 2015	1.JPS_trunk F_sagital(error:deg)-	1.sym index of ext abd obliq(0-0.5)-	0.589	0.110	0.846	0.019
axial seg	Ryerson 2008	1.JPS_trunk_Horizontal(error:deg)-	1.BBS(loss-intact:0-56)	0.480	0.048	0.761	0.031
axial seg				0.443	0.211	0.628	0.000
lower limb	Fujita 2020	1.SIAS_Position_toe(loss-intact:0-3)	1.BI-Walking (dep/indep)	0.250	0.064	0.419	0.009
lower limb	Gorst 2019	1.rNSA-Proprio_distal(loss-intact:0-8)	1.TUG(time:s)-	0.120	-0.037	0.272	0.135
lower limb	Lin 2005	1.JPS_ankle(error:deg)-	1.FMA-LE(loss-intact:0-34)	0.228	-0.226	0.601	0.325
lower limb	Niam 1999	1.JPS_Ankle(impaired-intact:0-1)	1.BBS(loss-intact:0-56)	0.377	0.019	0.649	0.039
lower limb	Perry 1995	1.JPS_Ankle/knee/hip(abs-norm:0-3)	1.walk handicap(physio-commu:1-6)	0.000	-0.162	0.162	1.000
lower limb	Soyuer 2007	1.JPS_ankle(Error:deg)-	1.Falls_faller(non-repeated:0-3)	0.169	-0.028	0.354	0.092
lower limb	Tsang 2013	1.JPS_knee(Error:deg)-	1.Pointing(accuracy:mm)	0.000	-0.512	0.512	1.000
lower limb	Vlaar 2017	1.rNSA-Proprio_wrist(abs-intact:0-2)	1.FMA-UE(loss-intact:0-66)	0.481	0.146	0.717	0.006
lower limb				0.174	0.068	0.276	0.001
upper limb	Borstad 2016	1.Reach target(error dist:cm)-	1.BBT(no. of blocks)	0.770	0.351	0.932	0.002
upper limb	Cherpin 2019	1.Arm push(error dist:cm)-	1.FMA-UE(loss-intact:0-66)	0.342	-0.118	0.681	0.142
upper limb	Cho 2014	1.Hiting target(Totl error dist:cm)-	1.FMA-UE(loss-intact:0-66)	0.710	0.145	0.926	0.019
upper limb	dos Santos 2015	1.JPS_SH Abd(absol error:deg)-	1.FMA-UE(loss-intact:0-66)	0.000	-0.551	0.551	1.000
upper limb	Dukelow 2012	1.TLT(intact-severe deficit:0-3)-	1.MAS(norm-severe:0-4)-	0.000	-0.206	0.206	1.000
upper limb	Kantak 2016	1.Elbowmatch task(error:deg)-	1.Reach-pick time sym(s)-	0.618	0.130	0.865	0.017
upper limb	Kenzie 2016	1.TLT(intact-severe defict:0-3)-	1.FIM(Totl dep-indep:18-126)	0.360	0.208	0.495	0.000
upper limb	Leibowitz 2008	1.Hand match(distance error:cm)-	1.Motor deficit(intact-max disab:0-3)-	0.097	-0.338	0.498	0.671
upper limb	Mercier 2004	1.FMA-UL proprio(loss-intact:0-2)	1.MAS(norm-severe:0-4)-	0.286	-0.244	0.685	0.289
upper limb	Meyer 2016	1.TLT(intact-severe defict:0-3)-	1.FMA-UE(loss-intact:0-66)	0.369	0.205	0.513	0.000
upper limb	Mochizuki 2019	1.Arm position(Shift:cm)-	1.MAS(norm-severe:0-4)-	0.096	-0.142	0.324	0.431
upper limb	Rand 2018	1.TLT(intact-severe defict:0-3)-	1.FMA-UE(loss-intact:0-66)	0.410	0.234	0.560	0.000
upper limb	Rand 2018(2)	1.TLT(intact-severe defict:0-3)-	1.FIM(Totl dep-indep:18-126)	0.402	0.173	0.589	0.001
upper limb	Smith 1983	1.TLT(impaired-intact:0-1)	1.Strength_UE(loss-no-loss:0-1)	0.222	0.091	0.344	0.001
upper limb	Wagner 2006	1.JPS_1stMCP(abs-intact:0-1)	1.MAS(norm-severe:0-4)-	0.320	0.033	0.558	0.030
upper limb	Wu 2016	1.rNSA-Proprio(abs-intact:0-2)	1.FMA-UE(loss-intact:0-66)	0.220	0.060	0.369	0.007
upper limb	Zbytniewska 2021	1.JPS_2ndMCP(Error:deg)-	1.FMA-UE(loss-intact:0-66)	0.320	-0.046	0.610	0.085
upper limb				0.292	0.209	0.370	0.000
Overall				0.258	0.196	0.319	0.000

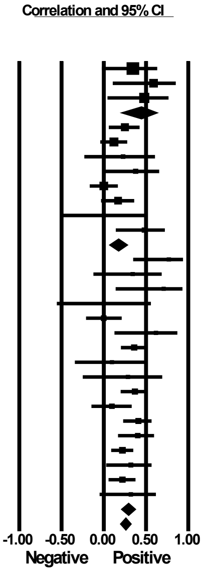

(B) Random-effect model of analysis: I^2=0%, 43%*, 33% across studies measuring proprioception in axial segment, upper limbs and lower limbs respectively. (*:p<0.05)

Group by 3. Matching side	Study name	Proprioception test	Motor function test	Statistics for each study: Correlation	Lower limit	Upper limit	p-Value
contralateral	Dukelow 2012	1.TLT(intact-severe deficit:0-3)-	1.MAS(norm-severe:0-4)-	0.000	-0.206	0.206	1.000
contralateral	Fujita 2020	1.SIAS_Position_toe(loss-intact:0-3)	1.BI-Walking (dep/indep)	0.250	0.064	0.419	0.009
contralateral	Gorst 2019	1.rNSA-Proprio_distal(loss-intact:0-8)	1.TUG(time:s)-	0.120	-0.037	0.272	0.135
contralateral	Kantak 2016	1.Elbowmatch task(error:deg)-	1.Reach-pick time sym(s)-	0.618	0.130	0.865	0.017
contralateral	Kenzie 2016	1.TLT(intact-severe defict:0-3)-	1.FIM(Totl dep-indep:18-126)	0.360	0.208	0.495	0.000
contralateral	Leibowitz 2008	1.Hand match(distance error:cm)-	1.Motor deficit(intact-max disab:0-3)-	0.097	-0.338	0.498	0.671
contralateral	Lin 2005	1.JPS_ankle(error:deg)-	1.FMA-LE(loss-intact:0-34)	0.228	-0.226	0.601	0.325
contralateral	Mercier 2004	1.FMA-UL proprio(loss-intact:0-2)	1.MAS(norm-severe:0-4)-	0.286	-0.244	0.685	0.289
contralateral	Meyer 2016	1.TLT(intact-severe defict:0-3)-	1.FMA-UE(loss-intact:0-66)	0.369	0.205	0.513	0.000
contralateral	Mochizuki 2019	1.Arm position(Shift:cm)-	1.MAS(norm-severe:0-4)-	0.096	-0.142	0.324	0.431
contralateral	Perry 1995	1.JPS_Ankle/knee/hip(abs-norm:0-3)	1.walk handicap(physio-commu:1-6)	0.000	-0.162	0.162	1.000
contralateral	Rand 2018	1.TLT(intact-severe defict:0-3)-	1.FMA-UE(loss-intact:0-66)	0.410	0.234	0.560	0.000
contralateral	Rand 2018(2)	1.TLT(intact-severe defict:0-3)-	1.FIM(Totl dep-indep:18-126)	0.402	0.173	0.589	0.001
contralateral	Ryerson 2008	1.JPS_trunk_Horizontal(error:deg)-	1.BBS(loss-intact:0-56)	0.480	0.048	0.761	0.031
contralateral	Smith 1983	1.TLT(impaired-intact:0-1)	1.Strength_UE(loss-no-loss:0-1)	0.222	0.091	0.344	0.001
contralateral	Soyuer 2007	1.JPS_ankle(Error:deg)-	1.Falls_faller(non-repeated:0-3)	0.169	-0.028	0.354	0.092
contralateral	Tsang 2013	1.JPS_knee(Error:deg)-	1.Pointing(accuracy:mm)	0.000	-0.512	0.512	1.000
contralateral	Vlaar 2017	1.rNSA-Proprio_wrist(abs-intact:0-2)	1.FMA-UE(loss-intact:0-66)	0.481	0.146	0.717	0.006
contralateral	Wagner 2006	1.JPS_1stMCP(abs-intact:0-1)	1.MAS(norm-severe:0-4)-	0.320	0.033	0.558	0.030
contralateral	Wu 2016	1.rNSA-Proprio(abs-intact:0-2)	1.FMA-UE(loss-intact:0-66)	0.220	0.060	0.369	0.007
contralateral				0.240	0.170	0.308	0.000
ipsilateral	Bonan 2015	1.OKS stand_COP sway(mm)-	1.BBS(loss-intact:0-56)	0.344	-0.019	0.627	0.062
ipsilateral	Borstad 2016	1.Reach target(error dist:cm)-	1.BBT(no. of blocks)	0.770	0.351	0.932	0.002
ipsilateral	Cherpin 2019	1.Arm push(error dist:cm)-	1.FMA-UE(loss-intact:0-66)	0.342	-0.118	0.681	0.142
ipsilateral	Cho 2014	1.Hiting target(Totl error dist:cm)-	1.FMA-UE(loss-intact:0-66)	0.710	0.145	0.926	0.019
ipsilateral	dos Santos 2015	1.JPS_SH Abd(absol error:deg)-	1.FMA-UE(loss-intact:0-66)	0.000	-0.551	0.551	1.000
ipsilateral	Liao 2015	1.JPS_trunk F_sagital(error:deg)-	1.sym index of ext abd obliq(0-0.5)-	0.589	0.110	0.846	0.019
ipsilateral	Niam 1999	1.JPS_Ankle(impaired-intact:0-1)	1.BBS(loss-intact:0-56)	0.377	0.019	0.649	0.039
ipsilateral	Zbytniewska 2021	1.JPS_2ndMCP(Error:deg)-	1.FMA-UE(loss-intact:0-66)	0.320	-0.046	0.610	0.085
ipsilateral				0.412	0.254	0.548	0.000
Overall				0.267	0.202	0.329	0.000

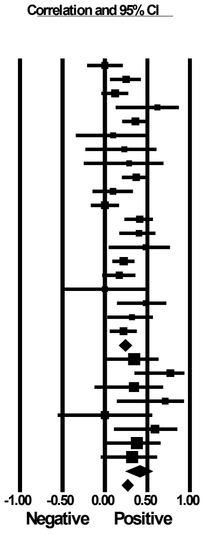

(C) Random-effect model of analysis: I^2=47%*, 9% across studies measuring proprioception in contralateral matching and ipsilateral matching respectively. (*:p<0.05)

Group by 4. Matching mov	Study name	Proprioception test	Motor function test	Statistics for each study: Correlation	Lower limit	Upper limit	p-Value
Active-Active	Bonan 2015	1.OKS stand_COP sway(mm)-	1.BBS(loss-intact:0-56)	0.344	-0.019	0.627	0.062
Active-Active	Cho 2014	1.Hiting target(Totl error dist:cm)-	1.FMA-UE(loss-intact:0-66)	0.710	0.145	0.926	0.019
Active-Active	Liao 2015	1.JPS_trunk F_sagital(error:deg)-	1.sym index of ext abd obliq(0-0.5)-	0.589	0.110	0.846	0.019
Active-Active				0.479	0.229	0.670	0.000
Passive-Active	Borstad 2016	1.Reach target(error dist:cm)-	1.BBT(no. of blocks)	0.770	0.351	0.932	0.002
Passive-Active	Cherpin 2019	1.Arm push(error dist:cm)-	1.FMA-UE(loss-intact:0-66)	0.342	-0.118	0.681	0.142
Passive-Active	Dukelow 2012	1.TLT(intact-severe deficit:0-3)-	1.MAS(norm-severe:0-4)-	0.000	-0.206	0.206	1.000
Passive-Active	Fujita 2020	1.SIAS_Position_toe(loss-intact:0-3)	1.BI-Walking (dep/indep)	0.250	0.064	0.419	0.009
Passive-Active	Gorst 2019	1.rNSA-Proprio_distal(loss-intact:0-8)	1.TUG(time:s)-	0.120	-0.037	0.272	0.135
Passive-Active	Kantak 2016	1.Elbowmatch task(error:deg)-	1.Reach-pick time sym(s)-	0.618	0.130	0.865	0.017
Passive-Active	Kenzie 2016	1.TLT(intact-severe defict:0-3)-	1.FIM(Totl dep-indep:18-126)	0.360	0.208	0.495	0.000
Passive-Active	Leibowitz 2008	1.Hand match(distance error:cm)-	1.Motor deficit(intact-max disab:0-3)-	0.097	-0.338	0.498	0.671
Passive-Active	Lin 2005	1.JPS_ankle(error:deg)-	1.FMA-LE(loss-intact:0-34)	0.228	-0.226	0.601	0.325
Passive-Active	Mercier 2004	1.FMA-UL proprio(loss-intact:0-2)	1.MAS(norm-severe:0-4)-	0.286	-0.244	0.685	0.289
Passive-Active	Meyer 2016	1.TLT(intact-severe defict:0-3)-	1.FMA-UE(loss-intact:0-66)	0.369	0.205	0.513	0.000
Passive-Active	Mochizuki 2019	1.Arm position(Shift:cm)-	1.MAS(norm-severe:0-4)-	0.096	-0.142	0.324	0.431
Passive-Active	Perry 1995	1.JPS_Ankle/knee/hip(abs-norm:0-3)	1.walk handicap(physio-commu:1-6)	0.000	-0.162	0.162	1.000
Passive-Active	Rand 2018	1.TLT(intact-severe defict:0-3)-	1.FMA-UE(loss-intact:0-66)	0.410	0.234	0.560	0.000
Passive-Active	Rand 2018(2)	1.TLT(intact-severe defict:0-3)-	1.FIM(Totl dep-indep:18-126)	0.402	0.173	0.589	0.001
Passive-Active	Ryerson 2008	1.JPS_trunk_Horizontal(error:deg)-	1.BBS(loss-intact:0-56)	0.480	0.048	0.761	0.031
Passive-Active	Smith 1983	1.TLT(impaired-intact:0-1)	1.Strength_UE(loss-no-loss:0-1)	0.222	0.091	0.344	0.001
Passive-Active	Soyuer 2007	1.JPS_ankle(Error:deg)-	1.Falls_faller(non-repeated:0-3)	0.169	-0.028	0.354	0.092
Passive-Active	Tsang 2013	1.JPS_knee(Error:deg)-	1.Pointing(accuracy:mm)	0.000	-0.512	0.512	1.000
Passive-Active	Vlaar 2017	1.rNSA-Proprio_wrist(abs-intact:0-2)	1.FMA-UE(loss-intact:0-66)	0.481	0.146	0.717	0.006
Passive-Active	Wagner 2006	1.JPS_1stMCP(abs-intact:0-1)	1.MAS(norm-severe:0-4)-	0.320	0.033	0.558	0.030
Passive-Active	Wu 2016	1.rNSA-Proprio(abs-intact:0-2)	1.FMA-UE(loss-intact:0-66)	0.220	0.060	0.369	0.007
Passive-Active				0.252	0.180	0.322	0.000
Passive-Passive	dos Santos 2015	1.JPS_SH Abd(absol error:deg)-	1.FMA-UE(loss-intact:0-66)	0.000	-0.551	0.551	1.000
Passive-Passive	Niam 1999	1.JPS_Ankle(impaired-intact:0-1)	1.BBS(loss-intact:0-56)	0.377	0.019	0.649	0.039
Passive-Passive				0.271	-0.078	0.561	0.126
Passive-Percep	Zbytniewska 2021	1.JPS_2ndMCP(Error:deg)-	1.FMA-UE(loss-intact:0-66)	0.320	-0.046	0.610	0.085
Passive-Percep				0.320	-0.046	0.610	0.085
Overall				0.270	0.204	0.335	0.000

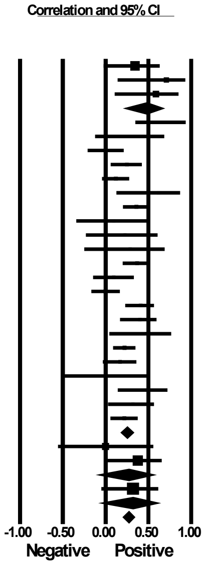

(D) Random-effect model of analysis: I^2=0%, 50%*, 13%, 0% across studies measuring proprioception in different movement modes respectively. (*:p<0.05)

Group by<br>5. Proprio Jt plane	Study name	Proprioception test	Motor function test	Correlation	Lower limit	Upper limit	p-Value
				Statistics for each study			
multiple	Bonan 2015	1.OKS stand_COP sway(mm)-	1.BBS(loss-intact:0-56)	0.344	-0.019	0.627	0.062
multiple	Borstad 2016	1.Reach target(error dist:cm)-	1.BBT(no. of blocks)	0.770	0.351	0.932	0.002
multiple	Cherpin 2019	1.Arm push(error dist:cm)-	1.FMA-UE(loss-intact:0-66)	0.342	-0.118	0.681	0.142
multiple	Cho 2014	1.Hiting target(Totl error dist:cm)-	1.FMA-UE(loss-intact:0-66)	0.710	0.145	0.926	0.019
multiple	Dukelow 2012	1.TLT(intact-severe deficit:0-3)-	1.MAS(norm-severe:0-4)-	0.000	-0.206	0.206	1.000
multiple	Kenzie 2016	1.TLT(intact-severe defict:0-3)-	1.FIM(Totl dep-indep:18-126)	0.360	0.208	0.495	0.000
multiple	Leibowitz 2008	1.Hand match(distance error:cm)-	1.Motor deficit(intact-max disab:0-3)-	0.097	-0.338	0.498	0.671
multiple	Liao 2015	1.JPS_trunk F_sagital(error:deg)-	1.sym index of ext abd obliq(0-0.5)-	0.589	0.110	0.846	0.019
multiple	Mochizuki 2019	1.Arm position(Shift:cm)-	1.MAS(norm-severe:0-4)-	0.096	-0.142	0.324	0.431
multiple	Rand 2018	1.TLT(intact-severe defict:0-3)-	1.FMA-UE(loss-intact:0-66)	0.410	0.234	0.560	0.000
multiple	Rand 2018(2)	1.TLT(intact-severe defict:0-3)-	1.FIM(Totl dep-indep:18-126)	0.402	0.173	0.589	0.001
multiple	Ryerson 2008	1.JPS_trunk_Horizontal(error:deg)-	1.BBS(loss-intact:0-56)	0.480	0.048	0.761	0.031
multiple	Smith 1983	1.TLT(impaired-intact:0-1)	1.Strength_UE(loss-no-loss:0-1)	0.222	0.091	0.344	0.001
multiple				0.315	0.204	0.418	0.000
single	dos Santos 2015	1.JPS_SH Abd(absol error:deg)-	1.FMA-UE(loss-intact:0-66)	0.000	-0.551	0.551	1.000
single	Fujita 2020	1.SIAS_Position_toe(loss-intact:0-3)	1.BI-Walking (dep/indep)	0.250	0.064	0.419	0.009
single	Gorst 2019	1.rNSA-Proprio_distal(loss-intact:0-8)	1.TUG(time:s)-	0.120	-0.037	0.272	0.135
single	Kantak 2016	1.Elbowmatch task(error:deg)-	1.Reach-pick time sym(s)-	0.618	0.130	0.865	0.017
single	Lin 2005	1.JPS_ankle(error:deg)-	1.FMA-LE(loss-intact:0-34)	0.228	-0.226	0.601	0.325
single	Mercier 2004	1.FMA-UL proprio(loss-intact:0-2)	1.MAS(norm-severe:0-4)-	0.286	-0.244	0.685	0.289
single	Meyer 2016	1.TLT(intact-severe defict:0-3)-	1.FMA-UE(loss-intact:0-66)	0.369	0.205	0.513	0.000
single	Niam 1999	1.JPS_Ankle(impaired-intact:0-1)	1.BBS(loss-intact:0-56)	0.377	0.019	0.649	0.039
single	Perry 1995	1.JPS_Ankle/knee/hip(abs-norm:0-3)	1.walk handicap(physio-commu:1-6)	0.000	-0.162	0.162	1.000
single	Soyuer 2007	1.JPS_ankle(Error:deg)-	1.Falls_faller(non-repeated:0-3)	0.169	-0.028	0.354	0.092
single	Tsang 2013	1.JPS_knee(Error:deg)-	1.Pointing(accuracy:mm)	0.000	-0.512	0.512	1.000
single	Vlaar 2017	1.rNSA-Proprio_wrist(abs-intact:0-2)	1.FMA-UE(loss-intact:0-66)	0.481	0.146	0.717	0.006
single	Wagner 2006	1.JPS_1stMCP(abs-intact:0-1)	1.MAS(norm-severe:0-4)-	0.320	0.033	0.558	0.030
single	Wu 2016	1.rNSA-Proprio(abs-intact:0-2)	1.FMA-UE(loss-intact:0-66)	0.220	0.060	0.369	0.007
single	Zbytniewska 2021	1.JPS_2ndMCP(Error:deg)-	1.FMA-UE(loss-intact:0-66)	0.320	-0.046	0.610	0.085
single				0.228	0.147	0.305	0.000
Overall				0.257	0.192	0.320	0.000

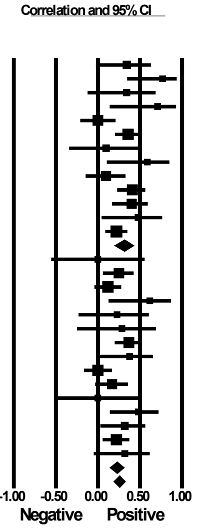

(E) Random-effect model of analysis: I^2=54%*, 31% across studies measuring proprioception in single and multiple joint planes respectively. (*:p<0.05)

Group by<br>6. Proprio data	Study name	Proprioception test	Motor function test	Correlation	Lower limit	Upper limit	p-Value
				Statistics for each study			
categorial	Smith 1983	1.TLT(impaired-intact:0-1)	1.Strength_UE(loss-no-loss:0-1)	0.222	0.091	0.344	0.001
categorial	Wagner 2006	1.JPS_1stMCP(abs-intact:0-1)	1.MAS(norm-severe:0-4)-	0.320	0.033	0.558	0.030
categorial				0.238	0.120	0.350	0.000
continous	Bonan 2015	1.OKS stand_COP sway(mm)-	1.BBS(loss-intact:0-56)	0.344	-0.019	0.627	0.062
continous	Borstad 2016	1.Reach target(error dist:cm)-	1.BBT(no. of blocks)	0.770	0.351	0.932	0.002
continous	Cherpin 2019	1.Arm push(error dist:cm)-	1.FMA-UE(loss-intact:0-66)	0.342	-0.118	0.681	0.142
continous	Cho 2014	1.Hiting target(Totl error dist:cm)-	1.FMA-UE(loss-intact:0-66)	0.710	0.145	0.926	0.019
continous	dos Santos 2015	1.JPS_SH Abd(absol error:deg)-	1.FMA-UE(loss-intact:0-66)	0.000	-0.551	0.551	1.000
continous	Kantak 2016	1.Elbowmatch task(error:deg)-	1.Reach-pick time sym(s)-	0.618	0.130	0.865	0.017
continous	Leibowitz 2008	1.Hand match(distance error:cm)-	1.Motor deficit(intact-max disab:0-3)-	0.097	-0.338	0.498	0.671
continous	Liao 2015	1.JPS_trunk F_sagital(error:deg)-	1.sym index of ext abd obliq(0-0.5)-	0.589	0.110	0.846	0.019
continous	Lin 2005	1.JPS_ankle(error:deg)-	1.FMA-LE(loss-intact:0-34)	0.228	-0.226	0.601	0.325
continous	Mochizuki 2019	1.Arm position(Shift:cm)-	1.MAS(norm-severe:0-4)-	0.096	-0.142	0.324	0.431
continous	Niam 1999	1.JPS_Ankle(impaired-intact:0-1)	1.BBS(loss-intact:0-56)	0.377	0.019	0.649	0.039
continous	Ryerson 2008	1.JPS_trunk_Horizontal(error:deg)-	1.BBS(loss-intact:0-56)	0.480	0.048	0.761	0.031
continous	Soyuer 2007	1.JPS_ankle(Error:deg)-	1.Falls_faller(non-repeated:0-3)	0.169	-0.028	0.354	0.092
continous	Tsang 2013	1.JPS_knee(Error:deg)-	1.Pointing(accuracy:mm)	0.000	-0.512	0.512	1.000
continous	Zbytniewska 2021	1.JPS_2ndMCP(Error:deg)-	1.FMA-UE(loss-intact:0-66)	0.320	-0.046	0.610	0.085
continous				0.304	0.184	0.414	0.000
ordinal	Dukelow 2012	1.TLT(intact-severe deficit:0-3)-	1.MAS(norm-severe:0-4)-	0.000	-0.206	0.206	1.000
ordinal	Fujita 2020	1.SIAS_Position_toe(loss-intact:0-3)	1.BI-Walking (dep/indep)	0.250	0.064	0.419	0.009
ordinal	Gorst 2019	1.rNSA-Proprio_distal(loss-intact:0-8)	1.TUG(time:s)-	0.120	-0.037	0.272	0.135
ordinal	Kenzie 2016	1.TLT(intact-severe defict:0-3)-	1.FIM(Totl dep-indep:18-126)	0.360	0.208	0.495	0.000
ordinal	Mercier 2004	1.FMA-UL proprio(loss-intact:0-2)	1.MAS(norm-severe:0-4)-	0.286	-0.244	0.685	0.289
ordinal	Meyer 2016	1.TLT(intact-severe defict:0-3)-	1.FMA-UE(loss-intact:0-66)	0.369	0.205	0.513	0.000
ordinal	Perry 1995	1.JPS_Ankle/knee/hip(abs-norm:0-3)	1.walk handicap(physio-commu:1-6)	0.000	-0.162	0.162	1.000
ordinal	Rand 2018	1.TLT(intact-severe defict:0-3)-	1.FMA-UE(loss-intact:0-66)	0.410	0.234	0.560	0.000
ordinal	Rand 2018(2)	1.TLT(intact-severe defict:0-3)-	1.FIM(Totl dep-indep:18-126)	0.402	0.173	0.589	0.001
ordinal	Vlaar 2017	1.rNSA-Proprio_wrist(abs-intact:0-2)	1.FMA-UE(loss-intact:0-66)	0.481	0.146	0.717	0.006
ordinal	Wu 2016	1.rNSA-Proprio(abs-intact:0-2)	1.FMA-UE(loss-intact:0-66)	0.220	0.060	0.369	0.007
ordinal				0.253	0.151	0.349	0.000
Overall				0.263	0.199	0.325	0.000

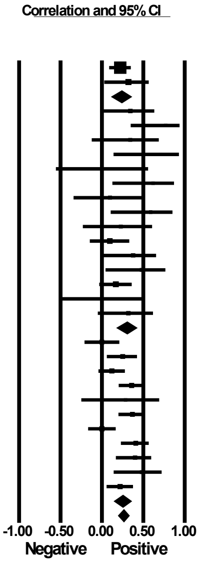

(F) Random-effect model of analysis: I^2=0%, 27%, 65%* across studies measuring proprioception with continuous, ordinal and categorial data as results respectively. (*:p<0.05)

	Study name	Proprioception test	Motor function test	Correlation	Lower limit	Upper limit	p-Value
1.1Function_tone	Dukelow 2012	1.TLT(intact-severe deficit:0-3)-	1.MAS(norm-severe:0-4)-	0.000	-0.206	0.206	1.000
1.1Function_tone	Mercier 2004	1.FMA-UL proprio(loss-intact:0-2)	1.MAS(norm-severe:0-4)-	0.286	-0.244	0.685	0.289
1.1Function_tone	Mochizuki 2019	1.Arm position(Shift:cm)-	1.MAS(norm-severe:0-4)-	0.096	-0.142	0.324	0.431
1.1Function_tone	Wagner 2006	1.JPS_1stMCP(abs-intact:0-1)	1.MAS(norm-severe:0-4)-	0.320	0.033	0.558	0.030
1.1Function_tone				0.116	-0.019	0.246	0.091
1.2Function_strength	Smith 1983	1.TLT(impaired-intact:0-1)	1.Strength_UE(loss-no-loss:0-1)	0.222	0.091	0.344	0.001
1.2Function_strength				0.222	0.091	0.344	0.001
1.3Function_movement	Cherpin 2019	1.Arm push(error dist:cm)-	1.FMA-UE(loss-intact:0-66)	0.342	-0.118	0.681	0.142
1.3Function_movement	Cho 2014	1.Hiting target(Totl error dist:cm)-	1.FMA-UE(loss-intact:0-66)	0.710	0.145	0.926	0.019
1.3Function_movement	dos Santos 2015	1.JPS_SH Abd(absol error:deg)-	1.FMA-UE(loss-intact:0-66)	0.000	-0.551	0.551	1.000
1.3Function_movement	Kantak 2016	1.Elbowmatch task(error:deg)-	1.Reach-pick time sym(s)-	0.618	0.130	0.865	0.017
1.3Function_movement	Liao 2015	1.JPS_trunk F_sagital(error:deg)-	1.sym index of ext abd obliq(0-0.5)-	0.589	0.110	0.846	0.019
1.3Function_movement	Lin 2005	1.JPS_ankle(error:deg)-	1.FMA-LE(loss-intact:0-34)	0.228	-0.226	0.601	0.325
1.3Function_movement	Meyer 2016	1.TLT(intact-severe defict:0-3)-	1.FMA-UE(loss-intact:0-66)	0.369	0.205	0.513	0.000
1.3Function_movement	Rand 2018	1.TLT(intact-severe defict:0-3)-	1.FMA-UE(loss-intact:0-66)	0.410	0.234	0.560	0.000
1.3Function_movement	Tsang 2013	1.JPS_knee(Error:deg)-	1.Pointing(accuracy:mm)	0.000	-0.512	0.512	1.000
1.3Function_movement	Vlaar 2017	1.rNSA-Proprio_wrist(abs-intact:0-2)	1.FMA-UE(loss-intact:0-66)	0.481	0.146	0.717	0.006
1.3Function_movement	Wu 2016	1.rNSA-Proprio(abs-intact:0-2)	1.FMA-UE(loss-intact:0-66)	0.220	0.060	0.369	0.007
1.3Function_movement	Zbytniewska 2021	1.JPS_2ndMCP(Error:deg)-	1.FMA-UE(loss-intact:0-66)	0.320	-0.046	0.610	0.085
1.3Function_movement				0.338	0.259	0.413	0.000
2.1Activity_perform	Bonan 2015	1.OKS stand_COP sway(mm)-	1.BBS(loss-intact:0-56)	0.344	-0.019	0.627	0.062
2.1Activity_perform	Borstad 2016	1.Reach target(error dist:cm)-	1.BBT(no. of blocks)	0.770	0.351	0.932	0.002
2.1Activity_perform	Gorst 2019	1.rNSA-Proprio_distal(loss-intact:0-8)	1.TUG(time:s)-	0.120	-0.037	0.272	0.135
2.1Activity_perform	Niam 1999	1.JPS_Ankle(impaired-intact:0-1)	1.BBS(loss-intact:0-56)	0.377	0.019	0.649	0.039
2.1Activity_perform	Ryerson 2008	1.JPS_trunk_Horizontal(error:deg)-	1.BBS(loss-intact:0-56)	0.480	0.048	0.761	0.031
2.1Activity_perform				0.239	0.115	0.356	0.000
2.2Activity_independ	Fujita 2020	1.SIAS_Position_toe(loss-intact:0-3)	1.BI-Walking (dep/indep)	0.250	0.064	0.419	0.009
2.2Activity_independ	Kenzie 2016	1.TLT(intact-severe defict:0-3)-	1.FIM(Totl dep-indep:18-126)	0.360	0.208	0.495	0.000
2.2Activity_independ	Leibowitz 2008	1.Hand match(distance error:cm)-	1.Motor deficit(intact-max disab:0-3)-	0.097	-0.338	0.498	0.671
2.2Activity_independ	Rand 2018(2)	1.TLT(intact-severe defict:0-3)-	1.FIM(Totl dep-indep:18-126)	0.402	0.173	0.589	0.001
2.2Activity_independ				0.319	0.218	0.413	0.000
3.Participation	Perry 1995	1.JPS_Ankle/knee/hip(abs-norm:0-3)	1.walk handicap(physio-commu:1-6)	0.000	-0.162	0.162	1.000
3.Participation				0.000	-0.162	0.162	1.000
4.Activity_perform_environ	Soyuer 2007	1.JPS_ankle(Error:deg)-	1.Falls_faller(non-repeated:0-3)	0.169	-0.028	0.354	0.092
4.Activity_perform_environ				0.169	-0.028	0.354	0.092
Overall				0.244	0.199	0.288	0.000

Statistics for each study

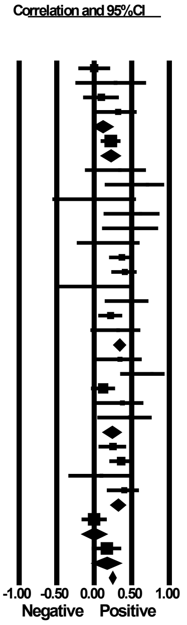

(G) Random-effect model of analysis: I^2=17%, 0%, 7%, 60%*, 0%, 0% across studies measuring motor function in ICF domains 1-4 respectively. (*:p<0.05)

Group by 8. Motor data	Study name	Proprioception test	Motor function test	Correlation	Lower limit	Upper limit	p-Value
categorial	Smith 1983	1.TLT(impaired-intact:0-1)	1.Strength_UE(loss-no-loss:0-1)	0.222	0.091	0.344	0.001
categorial	Soyuer 2007	1.JPS_ankle(Error:deg)-	1.Falls_faller(non-repeated:0-3)	0.169	-0.028	0.354	0.092
categorial				0.205	0.097	0.309	0.000
continous	Borstad 2016	1.Reach target(error dist:cm)-	1.BBT(no. of blocks)	0.770	0.351	0.932	0.002
continous	Gorst 2019	1.rNSA-Proprio_distal(loss-intact:0-8)	1.TUG(time:s)-	0.120	-0.037	0.272	0.135
continous	Kantak 2016	1.Elbowmatch task(error:deg)-	1.Reach-pick time sym(s)-	0.618	0.130	0.865	0.017
continous	Liao 2015	1.JPS_trunk F_sagital(error:deg)-	1.sym index of ext abd obliq(0-0.5)-	0.589	0.110	0.846	0.019
continous	Tsang 2013	1.JPS_knee(Error:deg)-	1.Pointing(accuracy:mm)	0.000	-0.512	0.512	1.000
continous				0.424	0.073	0.681	0.019
ordinal	Bonan 2015	1.OKS stand_COP sway(mm)-	1.BBS(loss-intact:0-56)	0.344	-0.019	0.627	0.062
ordinal	Cherpin 2019	1.Arm push(error dist:cm)-	1.FMA-UE(loss-intact:0-66)	0.342	-0.118	0.681	0.142
ordinal	Cho 2014	1.Hiting target(Totl error dist:cm)-	1.FMA-UE(loss-intact:0-66)	0.710	0.145	0.926	0.019
ordinal	dos Santos 2015	1.JPS_SH Abd(absol error:deg)-	1.FMA-UE(loss-intact:0-66)	0.000	-0.551	0.551	1.000
ordinal	Dukelow 2012	1.TLT(intact-severe deficit:0-3)-	1.MAS(norm-severe:0-4)-	0.000	-0.206	0.206	1.000
ordinal	Fujita 2020	1.SIAS_Position_toe(loss-intact:0-3)	1.BI-Walking (dep/indep)	0.250	0.064	0.419	0.009
ordinal	Kenzie 2016	1.TLT(intact-severe defict:0-3)-	1.FIM(Totl dep-indep:18-126)	0.360	0.208	0.495	0.000
ordinal	Leibowitz 2008	1.Hand match(distance error:cm)-	1.Motor deficit(intact-max disab:0-3)-	0.097	-0.338	0.498	0.671
ordinal	Lin 2005	1.JPS_ankle(error:deg)-	1.FMA-LE(loss-intact:0-34)	0.228	-0.226	0.601	0.325
ordinal	Mercier 2004	1.FMA-UL proprio(loss-intact:0-2)	1.MAS(norm-severe:0-4)-	0.286	-0.244	0.685	0.289
ordinal	Meyer 2016	1.TLT(intact-severe defict:0-3)-	1.FMA-UE(loss-intact:0-66)	0.369	0.205	0.513	0.000
ordinal	Mochizuki 2019	1.Arm position(Shift:cm)-	1.MAS(norm-severe:0-4)-	0.096	-0.142	0.324	0.431
ordinal	Niam 1999	1.JPS_Ankle(impaired-intact:0-1)	1.BBS(loss-intact:0-56)	0.377	0.019	0.649	0.039
ordinal	Perry 1995	1.JPS_Ankle/knee/hip(abs-norm:0-3)	1.walk handicap(physio-commu:1-6)	0.000	-0.162	0.162	1.000
ordinal	Rand 2018	1.TLT(intact-severe defict:0-3)-	1.FMA-UE(loss-intact:0-66)	0.410	0.234	0.560	0.000
ordinal	Rand 2018(2)	1.TLT(intact-severe defict:0-3)-	1.FIM(Totl dep-indep:18-126)	0.402	0.173	0.589	0.001
ordinal	Ryerson 2008	1.JPS_trunk_Horizontal(error:deg)-	1.BBS(loss-intact:0-56)	0.480	0.048	0.761	0.031
ordinal	Vlaar 2017	1.rNSA-Proprio_wrist(abs-intact:0-2)	1.FMA-UE(loss-intact:0-66)	0.481	0.146	0.717	0.006
ordinal	Wagner 2006	1.JPS_1stMCP(abs-intact:0-1)	1.MAS(norm-severe:0-4)-	0.320	0.033	0.558	0.030
ordinal	Wu 2016	1.rNSA-Proprio(abs-intact:0-2)	1.FMA-UE(loss-intact:0-66)	0.220	0.060	0.369	0.007
ordinal	Zbytniewska 2021	1.JPS_2ndMCP(Error:deg)-	1.FMA-UE(loss-intact:0-66)	0.320	-0.046	0.610	0.085
ordinal				0.272	0.195	0.345	0.000
Overall				0.254	0.193	0.314	0.000

Statistics for each study

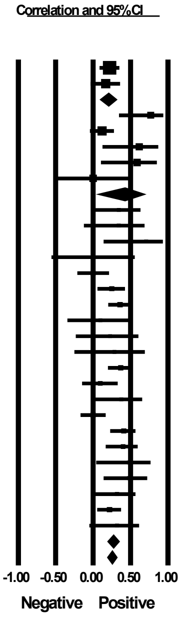

(H) Random-effect model of analysis: I^2=0%, 70%*, 42%* across studies measuring motor function with continuous, ordinal and categorial data as results respectively. (*:p<0.05)

Appendix I (full version for Figure 3): Subgroup analysis: association of proprioception with motor function after stroke. (A) Difference between proprioception subtypes measured in the tests (between-group difference: p=0.456). (B) Influence of body parts involved in the proprioception tests (between-group difference: p=0.055). (C) Influence of matching side involved in the proprioception tests (side involved) (between-group difference: p=0.050). (D) Influence of the movement modes involved in the proprioception tests (between-group

difference: p=0.380). (E) Influence of joint planes measured in the proprioception tests (between-group difference: p=0.205). (F) Influence of result acuity of the proprioception tests (between-group difference: p=0.710). (G) Influence of ICF motor function domains (between-group difference: p=0.003). (H) Influence of result acuity of the proprioception tests (between-group difference: p=0.364).
