# Supplementary material for: Correlation Between Proprioceptive Impairment and Motor Deficits After Stroke: A Meta-Analysis Review
Source: Front Neurol. 2022 Jan 13;12:688616. doi: 10.3389/fneur.2021.688616 (PMC8793362; doi:10.3389/fneur.2021.688616)
Supplement: Supplementary file 2 [file Data_Sheet_2.pdf]

Study name	Motor function test	Proprioception test	Correlation	Lower limit	Upper limit	p-Value
Cherpin 2019	1.FMA-UE(loss-intact:0-66)	1.Arm push(error dist:cm)-	0.342	-0.118	0.681	0.142
dos Santos 2015	1.FMA-UE(loss-intact:0-66)	1.JPS_SH Abd(absol error:deg)-	0.000	-0.551	0.551	1.000
Dukelow 2012	1.MAS(norm-severe:0-4)-	1.TLT(intact-severe deficit:0-3)-	0.000	-0.206	0.206	1.000
Fujita 2020	1.BI-Walking (dep/indep)	1.SIAS_Position_toe(loss-intact:0-3)	0.250	0.064	0.419	0.009
Gorst 2019	1.TUG(time:s)-	1.rNSA-Proprio_distal(loss-intact:0-8)	0.120	-0.037	0.272	0.135
Kenzie 2016	1.FIM(Totl dep-indep:18-126)	1.TLT(intact-severe defict:0-3)-	0.360	0.208	0.495	0.000
Leibowitz 2008	1.Motor deficit(intact-max disab:0-3)-	1.Hand match(distance error:cm)-	0.097	-0.338	0.498	0.671
Lin 2005	1.FMA-LE(loss-intact:0-34)	1.JPS_ankle(error:deg)-	0.228	-0.226	0.601	0.325
Meyer 2016	1.FMA-UE(loss-intact:0-66)	1.TLT(intact-severe defict:0-3)-	0.369	0.205	0.513	0.000
Mochizuki 2019	1.MAS(norm-severe:0-4)-	1.Arm position(Shift:cm)-	0.096	-0.142	0.324	0.431
Niam 1999	1.BBS(loss-intact:0-56)	1.JPS_Ankle(impaired-intact:0-1)	0.377	0.019	0.649	0.039
Perry 1995	1.walk handicap(physio-commu:1-6)	1.JPS_Ankle/knee/hip(abs-norm:0-3)	0.000	-0.162	0.162	1.000
Rand 2018	1.FMA-UE(loss-intact:0-66)	1.TLT(intact-severe defict:0-3)-	0.410	0.234	0.560	0.000
Rand 2018(2)	1.FIM(Totl dep-indep:18-126)	1.TLT(intact-severe defict:0-3)-	0.402	0.173	0.589	0.001
Ryerson 2008	1.BBS(loss-intact:0-56)	1.JPS_trunk_Horizontal(error:deg)-	0.480	0.048	0.761	0.031
Smith 1983	1.Strength_UE(loss-no-loss:0-1)	1.TLT(impaired-intact:0-1)	0.222	0.091	0.344	0.001
Soyuer 2007	1.Falls_faller(non-repeated:0-3)	1.JPS_ankle(Error:deg)-	0.169	-0.028	0.354	0.092
Vlaar 2017	1.FMA-UE(loss-intact:0-66)	1.rNSA-Proprio_wrist(abs-intact:0-2)	0.481	0.146	0.717	0.006
Wagner 2006	1.MAS(norm-severe:0-4)-	1.JPS_1stMCP(abs-intact:0-1)	0.320	0.033	0.558	0.030
Wu 2016	1.FMA-UE(loss-intact:0-66)	1.rNSA-Proprio(abs-intact:0-2)	0.220	0.060	0.369	0.007
Zbytniewska 2021	1.FMA-UE(loss-intact:0-66)	1.JPS_2ndMCP(Error:deg)-	0.320	-0.046	0.610	0.085
			0.241	0.176	0.305	0.000

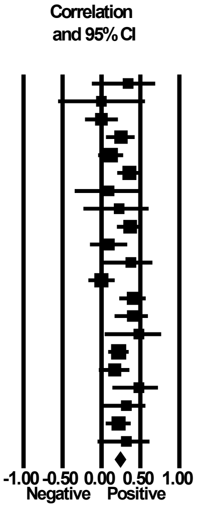

Appendix II. Meta-analysis on studies with a sample size of 20 and above: Association between proprioception and motor function after stroke.

Group by<br>1.Proprio subtype	Study name	Motor function test	Proprioception test	Correlation	Lower limit	Upper limit	p-Value
position	Cherpin 2019	1.FMA-UE(loss-intact:0-66)	1.Arm push(error dist:cm)-	0.342	-0.118	0.681	0.142
position	dos Santos 2015	1.FMA-UE(loss-intact:0-66)	1.JPS_SH Abd(absol error:deg)-	0.000	-0.551	0.551	1.000
position	Dukelow 2012	1.MAS(norm-severe:0-4)-	1.TLT(intact-severe deficit:0-3)-	0.000	-0.206	0.206	1.000
position	Kenzie 2016	1.FIM(Totl dep-indep:18-126)	1.TLT(intact-severe defict:0-3)-	0.360	0.208	0.495	0.000
position	Leibowitz 2008	1.Motor deficit(intact-max disab:0-3)-	1.Hand match(distance error:cm)-	0.097	-0.338	0.498	0.671
position	Lin 2005	1.FMA-LE(loss-intact:0-34)	1.JPS_ankle(error:deg)-	0.228	-0.226	0.601	0.325
position	Meyer 2016	1.FMA-UE(loss-intact:0-66)	1.TLT(intact-severe defict:0-3)-	0.369	0.205	0.513	0.000
position	Mochizuki 2019	1.MAS(norm-severe:0-4)-	1.Arm position(Shift:cm)-	0.096	-0.142	0.324	0.431
position	Niam 1999	1.BBS(loss-intact:0-56)	1.JPS_Ankle(impaired-intact:0-1)	0.377	0.019	0.649	0.039
position	Rand 2018	1.FMA-UE(loss-intact:0-66)	1.TLT(intact-severe defict:0-3)-	0.410	0.234	0.560	0.000
position	Rand 2018(2)	1.FIM(Totl dep-indep:18-126)	1.TLT(intact-severe defict:0-3)-	0.402	0.173	0.589	0.001
position	Ryerson 2008	1.BBS(loss-intact:0-56)	1.JPS_trunk_Horizontal(error:deg)-	0.480	0.048	0.761	0.031
position	Smith 1983	1.Strength_UE(loss-no-loss:0-1)	1.TLT(impaired-intact:0-1)	0.222	0.091	0.344	0.001
position	Soyuer 2007	1.Falls_faller(non-repeated:0-3)	1.JPS_ankle(Error:deg)-	0.169	-0.028	0.354	0.092
position	Wagner 2006	1.MAS(norm-severe:0-4)-	1.JPS_1stMCP(abs-intact:0-1)	0.320	0.033	0.558	0.030
position	Wu 2016	1.FMA-UE(loss-intact:0-66)	1.rNSA-Proprio(abs-intact:0-2)	0.220	0.060	0.369	0.007
position	Zbytniewska 2021	1.FMA-UE(loss-intact:0-66)	1.JPS_2ndMCP(Error:deg)-	0.320	-0.046	0.610	0.085
position				0.265	0.201	0.327	0.000
Position&motion	Fujita 2020	1.BI-Walking (dep/indep)	1.SIAS_Position_toe(loss-intact:0-3)	0.250	0.064	0.419	0.009
Position&motion	Gorst 2019	1.TUG(time:s)-	1.rNSA-Proprio_distal(loss-intact:0-8)	0.120	-0.037	0.272	0.135
Position&motion	Perry 1995	1.walk handicap(physio-commu:1-6)	1.JPS_Ankle/knee/hip(abs-norm:0-3)	0.000	-0.162	0.162	1.000
Position&motion	Vlaar 2017	1.FMA-UE(loss-intact:0-66)	1.rNSA-Proprio_wrist(abs-intact:0-2)	0.481	0.146	0.717	0.006
Position&motion				0.173	0.008	0.329	0.040
Overall				0.252	0.192	0.310	0.000

Correlation and 95% CI

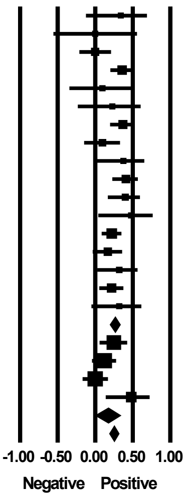

Group by<br>2. Proprio local	Study name	Motor function test	Proprioception test	Correlation	Lower limit	Upper limit	p-Value
axial seg	Ryerson 2008	1.BBS(loss-intact:0-56)	1.JPS_trunk_Horizontal(error:deg)-	0.480	0.048	0.761	0.031
axial seg				0.480	0.048	0.761	0.031
lower limb	Fujita 2020	1.BI-Walking (dep/indep)	1.SIAS_Position_toe(loss-intact:0-3)	0.250	0.064	0.419	0.009
lower limb	Gorst 2019	1.TUG(time:s)-	1.rNSA-Proprio_distal(loss-intact:0-8)	0.120	-0.037	0.272	0.135
lower limb	Lin 2005	1.FMA-LE(loss-intact:0-34)	1.JPS_ankle(error:deg)-	0.228	-0.226	0.601	0.325
lower limb	Niam 1999	1.BBS(loss-intact:0-56)	1.JPS_Ankle(impaired-intact:0-1)	0.377	0.019	0.649	0.039
lower limb	Perry 1995	1.walk handicap(physio-commu:1-6)	1.JPS_Ankle/knee/hip(abs-norm:0-3)	0.000	-0.162	0.162	1.000
lower limb	Soyuer 2007	1.Falls_faller(non-repeated:0-3)	1.JPS_ankle(Error:deg)-	0.169	-0.028	0.354	0.092
lower limb	Vlaar 2017	1.FMA-UE(loss-intact:0-66)	1.rNSA-Proprio_wrist(abs-intact:0-2)	0.481	0.146	0.717	0.006
lower limb				0.158	0.077	0.237	0.000
upper limb	Cherpin 2019	1.FMA-UE(loss-intact:0-66)	1.Arm push(error dist:cm)-	0.342	-0.118	0.681	0.142
upper limb	dos Santos 2015	1.FMA-UE(loss-intact:0-66)	1.JPS_SH Abd(absol error:deg)-	0.000	-0.551	0.551	1.000
upper limb	Dukelow 2012	1.MAS(norm-severe:0-4)-	1.TLT(intact-severe deficit:0-3)-	0.000	-0.206	0.206	1.000
upper limb	Kenzie 2016	1.FIM(Totl dep-indep:18-126)	1.TLT(intact-severe defict:0-3)-	0.360	0.208	0.495	0.000
upper limb	Leibowitz 2008	1.Motor deficit(intact-max disab:0-3)-	1.Hand match(distance error:cm)-	0.097	-0.338	0.498	0.671
upper limb	Meyer 2016	1.FMA-UE(loss-intact:0-66)	1.TLT(intact-severe defict:0-3)-	0.369	0.205	0.513	0.000
upper limb	Mochizuki 2019	1.MAS(norm-severe:0-4)-	1.Arm position(Shift:cm)-	0.096	-0.142	0.324	0.431
upper limb	Rand 2018	1.FMA-UE(loss-intact:0-66)	1.TLT(intact-severe defict:0-3)-	0.410	0.234	0.560	0.000
upper limb	Rand 2018(2)	1.FIM(Totl dep-indep:18-126)	1.TLT(intact-severe defict:0-3)-	0.402	0.173	0.589	0.001
upper limb	Smith 1983	1.Strength_UE(loss-no-loss:0-1)	1.TLT(impaired-intact:0-1)	0.222	0.091	0.344	0.001
upper limb	Wagner 2006	1.MAS(norm-severe:0-4)-	1.JPS_1stMCP(abs-intact:0-1)	0.320	0.033	0.558	0.030
upper limb	Wu 2016	1.FMA-UE(loss-intact:0-66)	1.rNSA-Proprio(abs-intact:0-2)	0.220	0.060	0.369	0.007
upper limb	Zbytniewska 2021	1.FMA-UE(loss-intact:0-66)	1.JPS_2ndMCP(Error:deg)-	0.320	-0.046	0.610	0.085
upper limb				0.266	0.209	0.321	0.000
Overall				0.231	0.185	0.277	0.000

Correlation and 95% CI

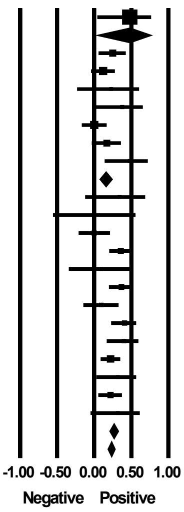

(B) Fixed-effect model of analysis: I^2=0%, 34%, 41% across studies measuring proprioception in axial segment, upper limbs and lower limbs respectively. ($p>0.05$)

Group by 3. Matching side	Study name	Motor function test	Proprioception test	Correlation	Lower limit	Upper limit	p-Value
contralateral	Dukelow 2012	1.MAS(norm-severe:0-4)-	1.TLT(intact-severe deficit:0-3)-	0.000	-0.206	0.206	1.000
contralateral	Fujita 2020	1.BI-Walking (dep/indep)	1.SIAS_Position_toe(loss-intact:0-3)	0.250	0.064	0.419	0.009
contralateral	Gorst 2019	1.TUG(time:s)-	1.rNSA-Proprio_distal(loss-intact:0-8)	0.120	-0.037	0.272	0.135
contralateral	Kenzie 2016	1.FIM(Totl dep-indep:18-126)	1.TLT(intact-severe defict:0-3)-	0.360	0.208	0.495	0.000
contralateral	Leibowitz 2008	1.Motor deficit(intact-max disab:0-3)-	1.Hand match(distance error:cm)-	0.097	-0.338	0.498	0.671
contralateral	Lin 2005	1.FMA-LE(loss-intact:0-34)	1.JPS_ankle(error:deg)-	0.228	-0.226	0.601	0.325
contralateral	Meyer 2016	1.FMA-UE(loss-intact:0-66)	1.TLT(intact-severe defict:0-3)-	0.369	0.205	0.513	0.000
contralateral	Mochizuki 2019	1.MAS(norm-severe:0-4)-	1.Arm position(Shift:cm)-	0.096	-0.142	0.324	0.431
contralateral	Perry 1995	1.walk handicap(physio-commu:1-6)	1.JPS_Ankle/knee/hip(abs-norm:0-3)	0.000	-0.162	0.162	1.000
contralateral	Rand 2018	1.FMA-UE(loss-intact:0-66)	1.TLT(intact-severe defict:0-3)-	0.410	0.234	0.560	0.000
contralateral	Rand 2018(2)	1.FIM(Totl dep-indep:18-126)	1.TLT(intact-severe defict:0-3)-	0.402	0.173	0.589	0.001
contralateral	Ryerson 2008	1.BBS(loss-intact:0-56)	1.JPS_trunk_Horizontal(error:deg)-	0.480	0.048	0.761	0.031
contralateral	Smith 1983	1.Strength_UE(loss-no-loss:0-1)	1.TLT(impaired-intact:0-1)	0.222	0.091	0.344	0.001
contralateral	Soyuer 2007	1.Falls_faller(non-repeated:0-3)	1.JPS_ankle(Error:deg)-	0.169	-0.028	0.354	0.092
contralateral	Vlaar 2017	1.FMA-UE(loss-intact:0-66)	1.rNSA-Proprio_wrist(abs-intact:0-2)	0.481	0.146	0.717	0.006
contralateral	Wagner 2006	1.MAS(norm-severe:0-4)-	1.JPS_1stMCP(abs-intact:0-1)	0.320	0.033	0.558	0.030
contralateral	Wu 2016	1.FMA-UE(loss-intact:0-66)	1.rNSA-Proprio(abs-intact:0-2)	0.220	0.060	0.369	0.007
contralateral				0.237	0.164	0.307	0.000
ipsilateral	Cherpin 2019	1.FMA-UE(loss-intact:0-66)	1.Arm push(error dist:cm)-	0.342	-0.118	0.681	0.142
ipsilateral	dos Santos 2015	1.FMA-UE(loss-intact:0-66)	1.JPS_SH Abd(absol error:deg)-	0.000	-0.551	0.551	1.000
ipsilateral	Niam 1999	1.BBS(loss-intact:0-56)	1.JPS_Ankle(impaired-intact:0-1)	0.377	0.019	0.649	0.039
ipsilateral	Zbytniewska 2021	1.FMA-UE(loss-intact:0-66)	1.JPS_2ndMCP(Error:deg)-	0.320	-0.046	0.610	0.085
ipsilateral				0.307	0.099	0.489	0.004
Overall				0.244	0.176	0.310	0.000

Statistics for each study

Correlation and 95% CI

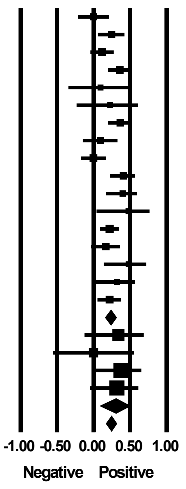

(C) Random-effect model of analysis: I^2=0%, 51%* across studies measuring proprioception in contralateral matching and ipsilateral matching respectively. (*:p<0.05)

Group by 4. Matching mov	Study name	Motor function test	Proprioception test	Correlation	Lower limit	Upper limit	p-Value
Passive-Active	Cherpin 2019	1.FMA-UE(loss-intact:0-66)	1.Arm push(error dist:cm)-	0.342	-0.118	0.681	0.142
Passive-Active	Dukelow 2012	1.MAS(norm-severe:0-4)-	1.TLT(intact-severe deficit:0-3)-	0.000	-0.206	0.206	1.000
Passive-Active	Fujita 2020	1.BI-Walking (dep/indep)	1.SIAS_Position_toe(loss-intact:0-3)	0.250	0.064	0.419	0.009
Passive-Active	Gorst 2019	1.TUG(time:s)-	1.rNSA-Proprio_distal(loss-intact:0-8)	0.120	-0.037	0.272	0.135
Passive-Active	Kenzie 2016	1.FIM(Totl dep-indep:18-126)	1.TLT(intact-severe defict:0-3)-	0.360	0.208	0.495	0.000
Passive-Active	Leibowitz 2008	1.Motor deficit(intact-max disab:0-3)-	1.Hand match(distance error:cm)-	0.097	-0.338	0.498	0.671
Passive-Active	Lin 2005	1.FMA-LE(loss-intact:0-34)	1.JPS_ankle(error:deg)-	0.228	-0.226	0.601	0.325
Passive-Active	Meyer 2016	1.FMA-UE(loss-intact:0-66)	1.TLT(intact-severe defict:0-3)-	0.369	0.205	0.513	0.000
Passive-Active	Mochizuki 2019	1.MAS(norm-severe:0-4)-	1.Arm position(Shift:cm)-	0.096	-0.142	0.324	0.431
Passive-Active	Perry 1995	1.walk handicap(physio-commu:1-6)	1.JPS_Ankle/knee/hip(abs-norm:0-3)	0.000	-0.162	0.162	1.000
Passive-Active	Rand 2018	1.FMA-UE(loss-intact:0-66)	1.TLT(intact-severe defict:0-3)-	0.410	0.234	0.560	0.000
Passive-Active	Rand 2018(2)	1.FIM(Totl dep-indep:18-126)	1.TLT(intact-severe defict:0-3)-	0.402	0.173	0.589	0.001
Passive-Active	Ryerson 2008	1.BBS(loss-intact:0-56)	1.JPS_trunk_Horizontal(error:deg)-	0.480	0.048	0.761	0.031
Passive-Active	Smith 1983	1.Strength_UE(loss-no-loss:0-1)	1.TLT(impaired-intact:0-1)	0.222	0.091	0.344	0.001
Passive-Active	Soyuer 2007	1.Falls_faller(non-repeated:0-3)	1.JPS_ankle(Error:deg)-	0.169	-0.028	0.354	0.092
Passive-Active	Vlaar 2017	1.FMA-UE(loss-intact:0-66)	1.rNSA-Proprio_wrist(abs-intact:0-2)	0.481	0.146	0.717	0.006
Passive-Active	Wagner 2006	1.MAS(norm-severe:0-4)-	1.JPS_1stMCP(abs-intact:0-1)	0.320	0.033	0.558	0.030
Passive-Active	Wu 2016	1.FMA-UE(loss-intact:0-66)	1.rNSA-Proprio(abs-intact:0-2)	0.220	0.060	0.369	0.007
Passive-Active				0.239	0.168	0.307	0.000
Passive-Passive	dos Santos 2015	1.FMA-UE(loss-intact:0-66)	1.JPS_SH Abd(absol error:deg)-	0.000	-0.551	0.551	1.000
Passive-Passive	Niam 1999	1.BBS(loss-intact:0-56)	1.JPS_Ankle(impaired-intact:0-1)	0.377	0.019	0.649	0.039
Passive-Passive				0.271	-0.078	0.561	0.126
Passive-Percep	Zbytniewska 2021	1.FMA-UE(loss-intact:0-66)	1.JPS_2ndMCP(Error:deg)-	0.320	-0.046	0.610	0.085
Passive-Percep				0.320	-0.046	0.610	0.085
Overall				0.243	0.175	0.308	0.000

Statistics for each study

Correlation and 95% CI

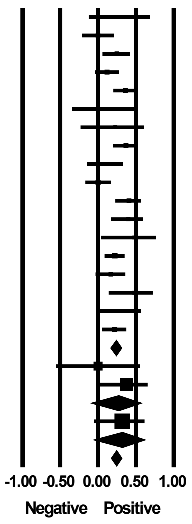

(D) Random-effect model of analysis: I^2=49%*, 13%, 0% across studies measuring proprioception in different movement modes respectively. (*:p<0.05)

Group by 5. Proprio Jt plane	Study name	Motor function test	Proprioception test	Correlation	Lower limit	Upper limit	p-Value
multiple	Cherpin 2019	1.FMA-UE(loss-intact:0-66)	1.Arm push(error dist:cm)-	0.342	-0.118	0.681	0.142
multiple	Dukelow 2012	1.MAS(norm-severe:0-4)-	1.TLT(intact-severe deficit:0-3)-	0.000	-0.206	0.206	1.000
multiple	Kenzie 2016	1.FIM(Totl dep-indep:18-126)	1.TLT(intact-severe defict:0-3)-	0.360	0.208	0.495	0.000
multiple	Leibowitz 2008	1.Motor deficit(intact-max disab:0-3)-	1.Hand match(distance error:cm)-	0.097	-0.338	0.498	0.671
multiple	Mochizuki 2019	1.MAS(norm-severe:0-4)-	1.Arm position(Shift:cm)-	0.096	-0.142	0.324	0.431
multiple	Rand 2018	1.FMA-UE(loss-intact:0-66)	1.TLT(intact-severe defict:0-3)-	0.410	0.234	0.560	0.000
multiple	Rand 2018(2)	1.FIM(Totl dep-indep:18-126)	1.TLT(intact-severe defict:0-3)-	0.402	0.173	0.589	0.001
multiple	Ryerson 2008	1.BBS(loss-intact:0-56)	1.JPS_trunk_Horizontal(error:deg)-	0.480	0.048	0.761	0.031
multiple	Smith 1983	1.Strength_UE(loss-no-loss:0-1)	1.TLT(impaired-intact:0-1)	0.222	0.091	0.344	0.001
multiple				0.264	0.154	0.367	0.000
single	dos Santos 2015	1.FMA-UE(loss-intact:0-66)	1.JPS_SH Abd(absol error:deg)-	0.000	-0.551	0.551	1.000
single	Fujita 2020	1.BI-Walking (dep/indep)	1.SIAS_Position_toe(loss-intact:0-3)	0.250	0.064	0.419	0.009
single	Gorst 2019	1.TUG(time:s)-	1.rNSA-Proprio_distal(loss-intact:0-8)	0.120	-0.037	0.272	0.135
single	Lin 2005	1.FMA-LE(loss-intact:0-34)	1.JPS_ankle(error:deg)-	0.228	-0.226	0.601	0.325
single	Meyer 2016	1.FMA-UE(loss-intact:0-66)	1.TLT(intact-severe defict:0-3)-	0.369	0.205	0.513	0.000
single	Niam 1999	1.BBS(loss-intact:0-56)	1.JPS_Ankle(impaired-intact:0-1)	0.377	0.019	0.649	0.039
single	Perry 1995	1.walk handicap(physio-commu:1-6)	1.JPS_Ankle/knee/hip(abs-norm:0-3)	0.000	-0.162	0.162	1.000
single	Soyuer 2007	1.Falls_faller(non-repeated:0-3)	1.JPS_ankle(Error:deg)-	0.169	-0.028	0.354	0.092
single	Vlaar 2017	1.FMA-UE(loss-intact:0-66)	1.rNSA-Proprio_wrist(abs-intact:0-2)	0.481	0.146	0.717	0.006
single	Wagner 2006	1.MAS(norm-severe:0-4)-	1.JPS_1stMCP(abs-intact:0-1)	0.320	0.033	0.558	0.030
single	Wu 2016	1.FMA-UE(loss-intact:0-66)	1.rNSA-Proprio(abs-intact:0-2)	0.220	0.060	0.369	0.007
single	Zbytniewska 2021	1.FMA-UE(loss-intact:0-66)	1.JPS_2ndMCP(Error:deg)-	0.320	-0.046	0.610	0.085
single				0.222	0.138	0.302	0.000
Overall				0.237	0.171	0.301	0.000

Statistics for each study

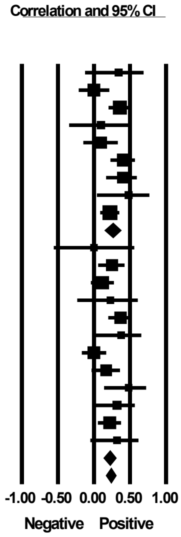

(E) Fixed-effect model of analysis: I^2=51%*, 35% across studies measuring proprioception in single and multiple joint planes respectively. (*p<0.05)

Group by 6. Proprio data	Study name	Motor function test	Proprioception test	Correlation	Lower limit	Upper limit	p-Value
categorial	Smith 1983	1.Strength_UE(loss-no-loss:0-1)	1.TLT(impaired-intact:0-1)	0.222	0.091	0.344	0.001
categorial	Wagner 2006	1.MAS(norm-severe:0-4)-	1.JPS_1stMCP(abs-intact:0-1)	0.320	0.033	0.558	0.030
categorial				0.238	0.120	0.350	0.000
continous	Cherpin 2019	1.FMA-UE(loss-intact:0-66)	1.Arm push(error dist:cm)-	0.342	-0.118	0.681	0.142
continous	dos Santos 2015	1.FMA-UE(loss-intact:0-66)	1.JPS_SH Abd(absol error:deg)-	0.000	-0.551	0.551	1.000
continous	Leibowitz 2008	1.Motor deficit(intact-max disab:0-3)-	1.Hand match(distance error:cm)-	0.097	-0.338	0.498	0.671
continous	Lin 2005	1.FMA-LE(loss-intact:0-34)	1.JPS_ankle(error:deg)-	0.228	-0.226	0.601	0.325
continous	Mochizuki 2019	1.MAS(norm-severe:0-4)-	1.Arm position(Shift:cm)-	0.096	-0.142	0.324	0.431
continous	Niam 1999	1.BBS(loss-intact:0-56)	1.JPS_Ankle(impaired-intact:0-1)	0.377	0.019	0.649	0.039
continous	Ryerson 2008	1.BBS(loss-intact:0-56)	1.JPS_trunk_Horizontal(error:deg)-	0.480	0.048	0.761	0.031
continous	Soyuer 2007	1.Falls_faller(non-repeated:0-3)	1.JPS_ankle(Error:deg)-	0.169	-0.028	0.354	0.092
continous	Zbytniewska 2021	1.FMA-UE(loss-intact:0-66)	1.JPS_2ndMCP(Error:deg)-	0.320	-0.046	0.610	0.085
continous				0.210	0.099	0.315	0.000
ordinal	Dukelow 2012	1.MAS(norm-severe:0-4)-	1.TLT(intact-severe deficit:0-3)-	0.000	-0.206	0.206	1.000
ordinal	Fujita 2020	1.BI-Walking (dep/indep)	1.SIAS_Position_toe(loss-intact:0-3)	0.250	0.064	0.419	0.009
ordinal	Gorst 2019	1.TUG(time:s)-	1.rNSA-Proprio_distal(loss-intact:0-8)	0.120	-0.037	0.272	0.135
ordinal	Kenzie 2016	1.FIM(Totl dep-indep:18-126)	1.TLT(intact-severe defict:0-3)-	0.360	0.208	0.495	0.000
ordinal	Meyer 2016	1.FMA-UE(loss-intact:0-66)	1.TLT(intact-severe defict:0-3)-	0.369	0.205	0.513	0.000
ordinal	Perry 1995	1.walk handicap(physio-commu:1-6)	1.JPS_Ankle/knee/hip(abs-norm:0-3)	0.000	-0.162	0.162	1.000
ordinal	Rand 2018	1.FMA-UE(loss-intact:0-66)	1.TLT(intact-severe defict:0-3)-	0.410	0.234	0.560	0.000
ordinal	Rand 2018(2)	1.FIM(Totl dep-indep:18-126)	1.TLT(intact-severe defict:0-3)-	0.402	0.173	0.589	0.001
ordinal	Vlaar 2017	1.FMA-UE(loss-intact:0-66)	1.rNSA-Proprio_wrist(abs-intact:0-2)	0.481	0.146	0.717	0.006
ordinal	Wu 2016	1.FMA-UE(loss-intact:0-66)	1.rNSA-Proprio(abs-intact:0-2)	0.220	0.060	0.369	0.007
ordinal				0.252	0.147	0.352	0.000
Overall				0.234	0.170	0.295	0.000

Statistics for each study

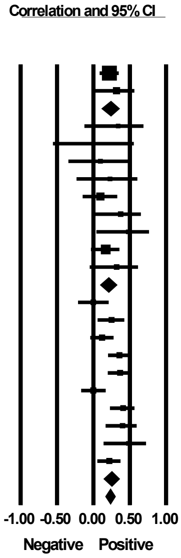

(F) Random-effect model of analysis: I^2=0%, 0%, 69%* across studies measuring proprioception with continuous, ordinal and categorial data as results respectively. (*:p<0.05)

Group by	Study name	Motor function test	Proprioception test	Correlation	Lower limit	Upper limit	p-Value
7. ICF domains							
1.1Function_tone	Dukelow 2012	1.MAS(norm-severe:0-4)-	1.TLT(intact-severe deficit:0-3)-	0.000	-0.206	0.206	1.000
1.1Function_tone	Mochizuki 2019	1.MAS(norm-severe:0-4)-	1.Arm position(Shift:cm)-	0.096	-0.142	0.324	0.431
1.1Function_tone	Wagner 2006	1.MAS(norm-severe:0-4)-	1.JPS_1stMCP(abs-intact:0-1)	0.320	0.033	0.558	0.030
1.1Function_tone				0.104	-0.035	0.239	0.141
1.2Function_strength	Smith 1983	1.Strength_UE(loss-no-loss:0-1)	1.TLT(impaired-intact:0-1)	0.222	0.091	0.344	0.001
1.2Function_strength				0.222	0.091	0.344	0.001
1.3Function_movement	Cherpin 2019	1.FMA-UE(loss-intact:0-66)	1.Arm push(error dist:cm)-	0.342	-0.118	0.681	0.142
1.3Function_movement	dos Santos 2015	1.FMA-UE(loss-intact:0-66)	1.JPS_SH Abd(absol error:deg)-	0.000	-0.551	0.551	1.000
1.3Function_movement	Lin 2005	1.FMA-LE(loss-intact:0-34)	1.JPS_ankle(error:deg)-	0.228	-0.226	0.601	0.325
1.3Function_movement	Meyer 2016	1.FMA-UE(loss-intact:0-66)	1.TLT(intact-severe defict:0-3)-	0.369	0.205	0.513	0.000
1.3Function_movement	Rand 2018	1.FMA-UE(loss-intact:0-66)	1.TLT(intact-severe defict:0-3)-	0.410	0.234	0.560	0.000
1.3Function_movement	Vlaar 2017	1.FMA-UE(loss-intact:0-66)	1.rNSA-Proprio_wrist(abs-intact:0-2)	0.481	0.146	0.717	0.006
1.3Function_movement	Wu 2016	1.FMA-UE(loss-intact:0-66)	1.rNSA-Proprio(abs-intact:0-2)	0.220	0.060	0.369	0.007
1.3Function_movement	Zbytniewska 2021	1.FMA-UE(loss-intact:0-66)	1.JPS_2ndMCP(Error:deg)-	0.320	-0.046	0.610	0.085
1.3Function_movement				0.324	0.240	0.403	0.000
2.1Activity_perform	Gorst 2019	1.TUG(time:s)-	1.rNSA-Proprio_distal(loss-intact:0-8)	0.120	-0.037	0.272	0.135
2.1Activity_perform	Niam 1999	1.BBS(loss-intact:0-56)	1.JPS_Ankle(impaired-intact:0-1)	0.377	0.019	0.649	0.039
2.1Activity_perform	Ryerson 2008	1.BBS(loss-intact:0-56)	1.JPS_trunk_Horizontal(error:deg)-	0.480	0.048	0.761	0.031
2.1Activity_perform				0.190	0.053	0.320	0.007
2.2Activity_independ	Fujita 2020	1.BI-Walking (dep/indep)	1.SIAS_Position_toe(loss-intact:0-3)	0.250	0.064	0.419	0.009
2.2Activity_independ	Kenzie 2016	1.FIM(Totl dep-indep:18-126)	1.TLT(intact-severe defict:0-3)-	0.360	0.208	0.495	0.000
2.2Activity_independ	Leibowitz 2008	1.Motor deficit(intact-max disab:0-3)-	1.Hand match(distance error:cm)-	0.097	-0.338	0.498	0.671
2.2Activity_independ	Rand 2018(2)	1.FIM(Totl dep-indep:18-126)	1.TLT(intact-severe defict:0-3)-	0.402	0.173	0.589	0.001
2.2Activity_independ				0.319	0.218	0.413	0.000
3.Participation	Perry 1995	1.walk handicap(physio-commu:1-6)	1.JPS_Ankle/knee/hip(abs-norm:0-3)	0.000	-0.162	0.162	1.000
3.Participation				0.000	-0.162	0.162	1.000
4.Activity_perform_environ	Soyuer 2007	1.Falls_faller(non-repeated:0-3)	1.JPS_ankle(Error:deg)-	0.169	-0.028	0.354	0.092
4.Activity_perform_environ				0.169	-0.028	0.354	0.092
Overall				0.231	0.185	0.277	0.000

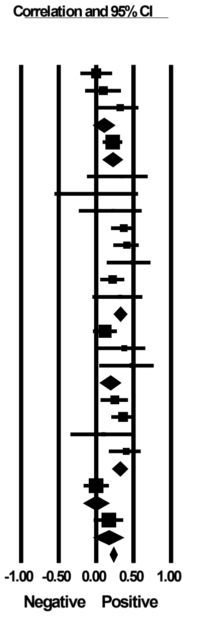

(G) Fixed-effect model of analysis: I^2=37%, 0%, 0%, 47%, 0%, 0% across studies measuring motor function in ICF domains 1-4 respectively. (p>0.05)

Group by	Study name	Proprioception test	Motor function test	Correlation	Lower limit	Upper limit	p-Value
8. Motor data							
categorial	Smith 1983	1.TLT(impaired-intact:0-1)	1.Strength_UE(loss-no-loss:0-1)	0.222	0.091	0.344	0.001
categorial	Soyuer 2007	1.JPS_ankle(Error:deg)-	1.Falls_faller(non-repeated:0-3)	0.169	-0.028	0.354	0.092
categorial				0.205	0.097	0.309	0.000
continous	Gorst 2019	1.rNSA-Proprio_distal(loss-intact:0-8)	1.TUG(time:s)-	0.120	-0.037	0.272	0.135
continous				0.120	-0.037	0.272	0.135
ordinal	Bonan 2015	1.OKS stand_COP sway(mm)-	1.BBS(loss-intact:0-56)	0.344	-0.019	0.627	0.062
ordinal	Cherpin 2019	1.Arm push(error dist:cm)-	1.FMA-UE(loss-intact:0-66)	0.342	-0.118	0.681	0.142
ordinal	Dukelow 2012	1.TLT(intact-severe deficit:0-3)-	1.MAS(norm-severe:0-4)-	0.000	-0.206	0.206	1.000
ordinal	Fujita 2020	1.SIAS_Position_toe(loss-intact:0-3)	1.BI-Walking (dep/indep)	0.250	0.064	0.419	0.009
ordinal	Kenzie 2016	1.TLT(intact-severe defict:0-3)-	1.FIM(Totl dep-indep:18-126)	0.360	0.208	0.495	0.000
ordinal	Leibowitz 2008	1.Hand match(distance error:cm)-	1.Motor deficit(intact-max disab:0-3)-	0.097	-0.338	0.498	0.671
ordinal	Lin 2005	1.JPS_ankle(error:deg)-	1.FMA-LE(loss-intact:0-34)	0.228	-0.226	0.601	0.325
ordinal	Meyer 2016	1.TLT(intact-severe defict:0-3)-	1.FMA-UE(loss-intact:0-66)	0.369	0.205	0.513	0.000
ordinal	Mochizuki 2019	1.Arm position(Shift:cm)-	1.MAS(norm-severe:0-4)-	0.096	-0.142	0.324	0.431
ordinal	Niam 1999	1.JPS_Ankle(impaired-intact:0-1)	1.BBS(loss-intact:0-56)	0.377	0.019	0.649	0.039
ordinal	Perry 1995	1.JPS_Ankle/knee/hip(abs-norm:0-3)	1.walk handicap(physio-commu:1-6)	0.000	-0.162	0.162	1.000
ordinal	Rand 2018	1.TLT(intact-severe defict:0-3)-	1.FMA-UE(loss-intact:0-66)	0.410	0.234	0.560	0.000
ordinal	Rand 2018(2)	1.TLT(intact-severe defict:0-3)-	1.FIM(Totl dep-indep:18-126)	0.402	0.173	0.589	0.001
ordinal	Ryerson 2008	1.JPS_trunk_Horizontal(error:deg)-	1.BBS(loss-intact:0-56)	0.480	0.048	0.761	0.031
ordinal	Vlaar 2017	1.rNSA-Proprio_wrist(abs-intact:0-2)	1.FMA-UE(loss-intact:0-66)	0.481	0.146	0.717	0.006
ordinal	Wagner 2006	1.JPS_1stMCP(abs-intact:0-1)	1.MAS(norm-severe:0-4)-	0.320	0.033	0.558	0.030
ordinal	Wu 2016	1.rNSA-Proprio(abs-intact:0-2)	1.FMA-UE(loss-intact:0-66)	0.220	0.060	0.369	0.007
ordinal	Zbytniewska 2021	1.JPS_2ndMCP(Error:deg)-	1.FMA-UE(loss-intact:0-66)	0.320	-0.046	0.610	0.085
ordinal				0.269	0.191	0.344	0.000
Overall				0.228	0.169	0.285	0.000

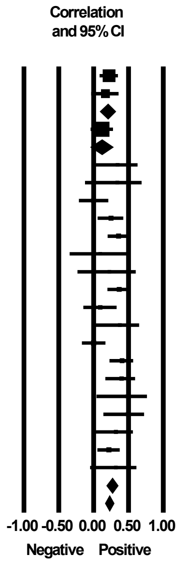

(H) Random-effect model of analysis: I^2=0%, 0%, 45%* across studies measuring motor function with continuous, ordinal and categorial data as results respectively. (*:p<0.05)

Appendix III. Subgroup analysis on studies with a sample size of 20 and above: association of proprioception with motor function after stroke. (A) Difference between proprioception subtypes measured in the tests (between-group difference: p > 0.05). (B) Influence of body parts involved in the proprioception tests (between-group difference: p > 0.05). (C) Influence of matching side involved (side involved) in the proprioception tests (between group difference: p > 0.05). (D) Influence of movement modes involved in the proprioception tests

(between group difference: $p > 0.05$). (E) Influence of joint planes measured in the proprioception tests (between-group difference: $p > 0.05$). (F) Influence of the result acuity of the proprioception tests (between-group difference: $p > 0.05$). (G) Influence of ICF motor function domains (between-group difference: $p < 0.05$). (H) Influence of the result acuity of the motor function tests (between-group difference: $p > 0.05$).
